# Artificial Intelligence, Clinical Decision Support Algorithms, Mathematical Models, Calculators Applications in Infertility: Systematic Review and Hands-On Digital Applications

**DOI:** 10.1016/j.mcpdig.2024.08.007

**Published:** 2024-08-26

**Authors:** Carlo Bulletti, Jason M. Franasiak, Andrea Busnelli, Romualdo Sciorio, Marco Berrettini, Lusine Aghajanova, Francesco M. Bulletti, Baris Ata

**Affiliations:** a Help Me Doctor, Assisted Reproductive Technology, Gynecological Endocrinology and Reproductive Surgery, Cattolica, Italy; b Department of Obstetrics, Gynecology, and Reproductive Science, Yale University, New Haven, CT; c IVI RMA, Reproductive Medicine Associates of New Jersey, NJ; d Department of Biomedical Sciences, Humanitas University, Milan, Italy; e IRCCS Humanitas Research Hospital, Rozzano, Italy; f Fertility Medicine and Gynaecological Endocrinology Unit, Department Woman Mother Child, Lausanne University Hospital, CHUV, Lausanne, Switzerland; g Department of Statistical Sciences, University of Bologna, Bologna, Italy; h Division of Reproductive Endocrinology and Infertility, Department of Obstetrics and Gynecology, Stanford University School of Medicine, Sunnyvale, CA; i Department Obstetrics and Gynecology, University Hospital of Vaud, Lausanne, Switzerland; j ART Fertility Clinics, Dubai, United Arab Emirates; k Department of Obstetrics and Gynecology, Koç University School of Medicine, Istanbul, Turkey

## Abstract

The aim of this systematic review was to identify clinical decision support algorithms (CDSAs) proposed for assisted reproductive technologies (ARTs) and to evaluate their effectiveness in improving ART cycles at every stage vs traditional methods, thereby providing an evidence-based guidance for their use in ART practice. A literature search on PubMed and Embase of articles published between 1 January 2013 and 31 January 2024 was performed to identify relevant articles. Prospective and retrospective studies in English on the use of CDSA for ART were included. Out of 1746 articles screened, 116 met the inclusion criteria. The selected articles were categorized into 3 areas: prognosis and patient counseling, clinical management, and embryo assessment. After screening, 11 CDSAs were identified as potentially valuable for clinical management and laboratory practices. Our findings highlight the potential of automated decision aids to improve in vitro fertilization outcomes. However, the main limitation of this review was the lack of standardization in validation methods across studies. Further validation and clinical trials are needed to establish the effectiveness of these tools in the clinical setting.

**Article Highlights**

• In recent years, several clinical decision support algorithms for assisted reproductive technologies have been proposed with the promise to improve treatment outcomes.
• Automated decision aids may be promising to enhance IVF outcomes, whereas the lack of standardization in validation methods across studies is a key limitation.

The global demand for assisted reproductive technologies (ARTs) has increased substantially, although delivery rates from these procedures remain moderate, with about 25% success per autologous embryo transfer and 30% for frozen-thawed transfers, according to the European IVF Monitoring Consortium for the European Society of Human Reproduction and Embryology.^1^ Despite this, cumulative live birth rates improve with multiple cycles, but repeated treatment and extended duration can exert emotional and financial strains.^2^^,^^3^

Assisted reproductive technology is a complex process, involving various techniques such as ovarian stimulation (OS), in vitro fertilization (IVF), and embryo cryopreservation. Recently, the use of clinical decision support algorithms (CDSAs) and artificial intelligence (AI), including machine learning (ML), has become prominent. These tools are designed to aid clinicians and patients in making informed decisions by managing and interpreting vast, complex data sets.^4^^,^^5^ When validated, they hold potential to enhance clinical prognosis, diagnosis, and treatment. Furthermore, they can empower patients in making informed decisions by reducing uncertainty, thereby fostering a new era of personalized medical care.^4^^,^^6^^,^^7^ However, the misuse or misinterpretation of these systems can lead to inappropriate interventions or misinformed patient expectations. Overall, despite the proliferation of such tools, their actual benefit in improving ART outcomes remains to be fully established.^6^^,^^8^, ^9^, ^10^ This review aimed to critically evaluate these tools and categorize them based on evidence.

## Methods

A systematic review was performed to identify CDSA, AI applications, algorithms, mathematical models, and calculators that have been proposed to support physicians and embryologists at different steps of ART, namely prediction of outcomes, patients profiling, treatment optimization and personalization, embryo evaluation, and laboratory management. The protocol for this systematic review has been registered with the international prospective register of systematic reviews PROSPERO (CRD42024499571). The systematic review was conducted according to the PRISMA guidelines.^11^

### Search Strategy

We searched PubMed and Embase for articles published from 1 January 2013 to 31 January 2024. Prospective and retrospective studies in English on the use of CDSA for ART were included. Specific keywords included the following: *assisted reproduction, medically assisted reproduction, in vitro fertilization, IVF, embryo culture, AI, ML, prediction models, automated algorithm, calculator, deep learning, automatic detection, automated data processing, time-lapse,* and *ultrasound*. Inclusion and exclusion criteria are documented with the patient/population, intervention, comparison and outcomes framework (Table 1). To supplement the database searches, we hand-searched bibliography of included articles along with screening similar articles.

**Table 1:** Patient/Population, Intervention, Comparison and Outcomes Model of the Systematic Review Conducted.

Population	Individuals or couples undergoing medically assisted reproduction procedures
Intervention	Computer-based decision aid systems designed to improve assisted reproductive technology outcomes at any stage
Comparison	Nonautomated systems (eg, human observation/nonautomated decision-making systems)
Outcomes	i. Implantation rate ii. Clinical pregnancy rate iii. Live birth rate
Study type	Inclusion criteria: interventional studies, prospective and retrospective studies, observational studies, and descriptive studies Exclusion criteria: case reports, editorials, letters to the editors, abstracts only
Date	From 1 January 2013 to 31 January 2024
Language	English

### Selection of Studies and Data Extraction

After automated deletion of duplicates, 2 researchers (C.B. and R.S.) independently screened titles and/or abstracts of the studies retrieved. Additional sources were screened to identify studies that potentially met the inclusion criteria. The full text of these potentially eligible studies was retrieved and independently assessed for eligibility by 2 members of the review team. Any disagreement between them over the eligibility of any study was resolved through discussion with a third reviewer (M.B.). Prospective and retrospective studies and randomized controlled trials were considered eligible for inclusion. Case reports and abstract only reports were excluded. Two collaborators (D.C. and D.F.) extracted the data using the standardized tables developed by the authors to ensure consistency. Studies comparing automated models vs humans were further evaluated for statistical significance. Results were considered significant when *P*<.05.

## Results

**Figure 1:**
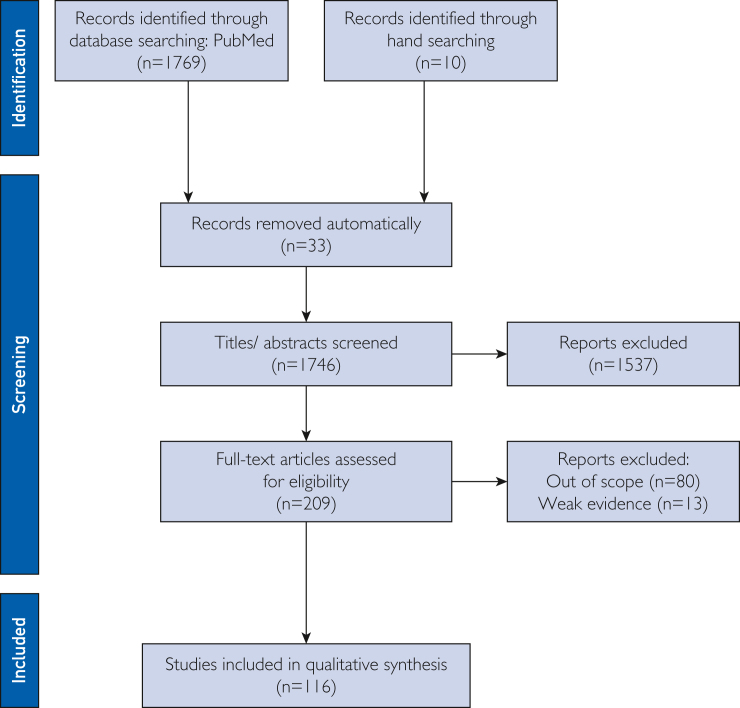
Study flow chart according to PRISMA guidelines.

The literature research identified 1746 articles that were further screened with the 2 progressive selection steps to identify articles that met the inclusion criteria (Figure 1). A total of 116 studies were included in the final analysis (Figure 1). Articles identified were divided according to the different stages of ART (Table 2).^6^^,^^8^, ^9^, ^10^^,^^12^, ^13^, ^14^, ^15^, ^16^, ^17^, ^18^, ^19^, ^20^, ^21^, ^22^, ^23^, ^24^, ^25^, ^26^, ^27^, ^28^, ^29^, ^30^, ^31^, ^32^, ^33^, ^34^, ^35^, ^36^, ^37^, ^38^, ^39^, ^40^, ^41^, ^42^, ^43^, ^44^, ^45^, ^46^, ^47^, ^48^, ^49^, ^50^, ^51^, ^52^, ^53^, ^54^, ^55^, ^56^, ^57^, ^58^, ^59^, ^60^, ^61^, ^62^, ^63^, ^64^, ^65^, ^66^, ^67^, ^68^, ^69^, ^70^, ^71^, ^72^, ^73^, ^74^, ^75^, ^76^, ^77^, ^78^, ^79^, ^80^, ^81^, ^82^, ^83^, ^84^, ^85^, ^86^, ^87^, ^88^, ^89^, ^90^, ^91^, ^92^, ^93^, ^94^, ^95^, ^96^, ^97^, ^98^, ^99^, ^100^, ^101^, ^102^, ^103^, ^104^, ^105^, ^106^, ^107^, ^108^, ^109^, ^110^, ^111^, ^112^, ^113^, ^114^, ^115^, ^116^, ^117^, ^118^, ^119^, ^120^, ^121^, ^122^, ^123^

**Table 2:** CDSA to Support for Prediction, Decision-Making, and Management of Patients at Different ART Stages.

CDSA	No. of articles identified	References
Prediction of treatment success based on patient characteristics/patient prognosis	51	6,8,10,12-59
Sperm analysis	11	43,60-69
Decision-making and counseling to support OS at various stages	9	8,9,32,70-75
Prediction of embryo quality/laboratory management and embryo selection	50	22,39,76-123

### Evidence in favor of CDSA in ART

We identified 11 automated tools that have reported efficacy for patient prognosis and counseling and clinical management as follows:.

• CDSA for patient prognosis and counseling:
  1. A noninvasive early presumption diagnosis method for pelvic endometriosis with a diagnostic penetrance of 90%.^12^
  2. A calculator to estimate the ovarian oocyte reserve based on the anti-Müllerian hormone (AMH), and/or antral follicle count, and based on previous response to OS able to identify patients with potential poor or suboptimal response to OS according to the Bologna or POSEIDON criteria.^13^^,^^124^
  3. Artificial intelligence for sperm count with evaluation of motility and morphology to classify patients as normal, hypospermic, and azoospermic based on published criteria^60^^,^^61^^,^^125^ and to assess DNA fragmentation.^126^
  4. A noninvasive tool to diagnose polycystic ovary syndrome (PCOS) based on body mass index (BMI, calculated as the weight in kilograms divided by the height in meters squared), upper limit of menstrual cycle length, serum AMH levels, and basal androstenedione levels.^14^
  5. A predictive model to estimate outcomes based on data from pretreatment (ie, before starting the first cycle of IVF) and posttreatment (ie, before starting the second cycle of IVF in those couples whose first complete cycle was unsuccessful).^6^
• CDSA for clinical management:
  6. A ML model to evaluate gonadotropin starting dose based on candidate’s characteristics.^9^^,^^70^
  7. A ML model to predict the day of oocyte trigger.^8^
  8. An algorithm to estimate the optimal number of oocytes to be fertilized based on the day of transfer, the number of viable blastocysts obtained, and the number of blastocysts needed to obtain 1 live birth.^15^
  9. A CDSA to manage clinical decisions regarding the following: (1) continuation of stimulation and (2) in case of discontinuation, whether to trigger or cancel the cycle or (3) in case of continuation of OS, whether to determine the days to follow-up and the need for dosage adjustment.^32^
  10. A calculator to estimate blastulation rate of metaphase II (MII) oocytes and to predict the chance of pregnancy.^40^
  11. A calculator of treatment success rates based on the patient’s age, the number of blastocysts to be transferred in sequence, and the preimplantation diagnosis of aneuploidies.^16^

All the above-mentioned CDSAs require further validation through prospective studies. However, the tools identified may be considered in the ART programs given the established benefit compared with human decision only.

### Automated Tools for ART

The implementation of predictive tools in ART is indeed expected to yield various positive outcomes. First, patients can expect more accurate prognostic predictions, thus setting more realistic expectations. Second, clinicians will benefit from streamlined decision-making processes leading to optimized treatment strategies and improved patient outcomes.

**Figure 2:**
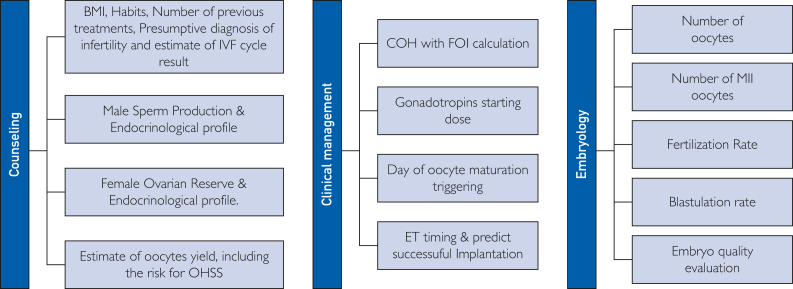
Steps of the infertile couple management journey for which clinical decision support algorithms have been proposed based on the findings of this review. BMI, body mass index; COH, controlled ovarian hyperstimulation; ET, embryo transfer; FOI, follicle-to-oocyte index; MII, metaphase II; OHSS, ovarian hyperstimulation syndrome.

The use of automated technologies in reproductive medicine is increasingly shaping how treatments are tailored and delivered (Table 2, Figure 2). Most of the studies identified evaluated the efficiency of automated models without direct comparison with human performance (Supplemental Table 1, available online at https://www.mcpdigitalhealth.org/), whereas a few made a direct comparison (Supplemental Table 2, available online at https://www.mcpdigitalhealth.org/). Further, we discuss the different tools and their advantages for each step of the ART journey. For each step, we also mention the studies that reported a significant advantage of automated tools vs humans.

1. Algorithms and AI in ART: Algorithms are crucial in ART for making treatment decisions by integrating various factors such as age, ovarian reserve, and genetic markers to recommend personalized strategies.^10^^,^^79^^,^^127^ AI systems further enhance this potential by analyzing extensive data to predict outcomes and continually refine treatment approaches through ML.
2. CDSA: These systems combine AI and clinical expertise to provide real-time decision support, helping clinicians to optimize treatment protocols based on comprehensive patient profiles.^4^
3. Predictive calculators: Using mathematical models, these calculators estimate the success likelihood of ART procedures, factoring in patient-specific variables such as age and BMI. This approach supports clinicians in creating more accurate and personalized treatment plans.

These technologies not only streamline clinical processes but also empower patients by providing clearer insights into their treatment options, enhancing the transparency and personalization of care in ART. This shift toward data-driven clinical decision-making will ultimately improve the accuracy and effectiveness of infertility treatments, thereby promising better outcomes for patients navigating these challenging processes.

### Tools for Patient Prognosis and Counseling

The integration of automated technologies in reproductive medicine has the potential to enhance treatment outcomes through personalized protocols and optimization of OS processes:

• Algorithms and calculators use patient-specific factors to predict treatment outcomes effectively. The Pregnancy Probability Calculator from the Institute for Reproductive Health at Georgetown University uses variables such as age, BMI, and diagnosis to predict pregnancy success.^128^
• Age-related predictive models estimate the chances of live birth based on age and other factors; for example, the Fertility Potential Calculator from the Society for Assisted Reproductive Technology.^129^
• Clinical conditions and diagnostic algorithms use comprehensive data to diagnose conditions like PCOS, using criteria such as the Rotterdam criteria.^14^^,^^17^

Predictive models in ART show potential in forecasting pregnancy and live birth rates but require larger, more diverse data sets for improved accuracy and generalizability. AI models analyze patient data such as age, hormone levels, and medical history to predict ART success, enabling customized treatment plans and realistic expectations for patients. However, these models are often limited by the homogeneity of the training data. Two studies evaluating the value of AI in predicting miscarriages and implantation rate reported significant improvement of AI prediction vs human prediction.^6^^,^^130^

AI algorithms can predict conditions such as endometriosis by analyzing questionnaire responses and various data sources with a 90% predictive capacity.^12^ Noninvasive diagnosis of conditions such as PCOS use AI algorithms analyze ultrasound images and hormonal profiles with higher accuracy and earlier than traditional methods.^14^

AI is also used to evaluate sperm parameters and DNA fragmentation, crucial for understanding male fertility issues and improving treatment plans. Tools such as the YO Home Sperm Test leverage smartphone technology for quick and accurate assessments.^60^^,^^61^^,^^125^^,^^126^ Despite these advancements, ethical concerns and the need for robust, transparent models remain significant challenges.

### AI and Algorithms to Optimize Clinical Management

Artificial intelligence has the potential to optimize personalized treatment plans by refining OS protocols and medication dosages, thereby enhancing ART outcomes. Additionally, wearable devices and AI applications facilitate continuous patient monitoring and timely interventions.

• Artificial intelligence in OS optimization tailor OS protocols by analyzing factors such as ovarian reserve markers and hormone levels to optimize the gonadotropin dosage and timing.^9^^,^^13^
• Mathematical models for ovarian response prediction predict responses based on variables such as AMH levels and antral follicle count, aiding in personalizing treatment plans and optimizing resource allocation.
• Algorithms for cycles personalization identify personalized medication dosages during OS enhance follicular development and minimize risks associated with poor or excessive responses.^131^

Leveraging AI in personalized IVF treatment plans can streamline the workflow in the clinics and offer transparency and realistic expectations for couples. Personalized treatment plans can not only maximize oocyte yield but also minimize the risk of ovarian hyperstimulation syndrome, and it can enhance scheduling precision for IVF cycles. However, the successful integration of these tools requires careful clinician oversight to ensure clinical appropriateness and address challenges related to data quality, interpretability, and generalizability.

### Automated Technologies in Embryo Assessment

In embryo selection, AI enhances the accuracy of identifying viable embryos by analyzing images and assessing quality and viability, although performance varies across clinics and patient populations. Tools such as Early Embryo Viability Assessment use AI to improve success rates by selecting the most viable embryos.

• Artificial intelligence in embryo quality assessment, like those developed by Kragh and Karstoft,^132^ enhance embryo selection by analyzing morphologic data to predict viability, improving accuracy and consistency in embryo selection.
• Time-lapse imaging applies mathematical models to time-lapse data to analyze morphokinetic parameters, aiding in embryo viability assessment and timing for transfer.^133^^,^^134^
• Artificial intelligence–assisted sperm analysis enhances the accuracy and efficiency of sperm analysis, which is crucial for selecting sperm with the best fertilization potential.

Although AI has not yet fully replaced human expertise in ART laboratories, its integration has significantly enhanced the precision and consistency of embryo quality assessments. Despite advancements, ongoing model training and validation are essential owing to subjective variability in embryo assessment and differing clinic practices. Continued research and development, along with the creation of standardized data sets, are essential for maximizing the potential of AI in this field.

### Automated Technologies for Patient Empowerment

The integration of AI, algorithms, and calculators into reproductive medicine facilitates a more tailored and efficient approach to treatment, potentially increasing the likelihood of success in ART procedures (Tables 2 and 3). The main advantages for the patients are as follows:

• Personalized treatment: Algorithms and calculators consider individual patient factors to customize treatment protocols, thereby increasing efficacy.
• Improved ovarian response: AI-assisted optimization leads to better oocyte yield and fewer adverse effects.
• Informed decision-making: Treatment outcome calculators provide patients with personalized prognostic information, aiding in informed decision-making and optimizing treatment planning.

**Table 3:** Predictive Calculators for IVF Programs.

Type of cycle	Calculator	Variables considered	Link
Patients using own gamete	SART IVF success estimator	Age, previous IVF cycles, and specific treatment details	https://w3.abdn.ac.uk/clsm/SARTIVF/tool/ivf1
	Boston IVF success rates calculator	Age, infertility diagnosis, and previous IVF outcomes	https://www.bostonivf.com/search/?q=donation
Donor IVF programs	SART donor egg calculator	Age of the donor, the recipient’s age, and other treatment variables	https://www.sartcorsonline.com/
	FertilityIQ donor egg IVF success	Age of the donor, the recipient’s age, and the number of embryos transferred	https://www.fertilityiq.com/
Gestational carrier IVF programs	SART gestational carrier IVF calculator	Gestational carrier’s age, the intended parent’s age, and treatment specifics	https://www.sartcorsonline.com/
	FertilityIQ gestational carrier IVF success rate calculator	Gestational carrier’s age, the intended parent’s age, and the number of embryos transferred	https://www.fertilityiq.com/

IVF, in vitro fertilization; SART, Society for Assisted Reproductive Technology.

Overall automated systems can improve transparent communication of predicted treatment outcomes, thereby providing patients with realistic expectations and aiding in informed decision-making. However, integrating AI in IVF programs has shown promise in enhancing fertility treatments. Clinicians must educate patients about AI’s role to improve transparency, manage expectations, and prevent dissatisfaction. Transparency ensures patients are aware of the technologies in their treatment, whereas informed consent requires patients to understand and agree to the use of AI tools, knowing their benefits and limitations. Proper education fosters trust in both the treatment process and the clinicians. Patients should be made aware that AI effectiveness depends on high-quality data, and although it can improve success probabilities, it does not guarantee positive outcomes. Patients should also be informed about data privacy and ethical considerations. Clinicians should explain how AI is applied at specific IVF stages, emphasizing that AI supports, not replaces, fertility specialists’ expertise.

Therefore, patients should receive comprehensive information about AI use in treatment. Questions should be encouraged and any concerns should be addressed. Providing brochures, videos, personalized sessions, and group discussions can help patients understand AI role in IVF and share experiences, ultimately building trust and confidence in using advanced technologies in fertility treatments.

### Implementation in the Clinics

Although these technologies offer significant advantages, their implementation in clinical settings requires careful validation to ensure accuracy and effectiveness. The integration of AI tools enhances clinical interventions when combined with human expertise. The synergy between AI capabilities and clinicians’ efforts leads to precise execution of each intervention step, transparent follow-up processes, and the development of detailed, strategic plans for achieving cumulative results over time.

Currently, AI-based diagnostics for assessing embryo quality and predicting pregnancy outcomes are not comparable with the accuracy of invasive prenatal diagnostics or noninvasive fetal evaluations conducted through human ultrasound. Furthermore, the use of AI raises concerns, particularly regarding the need to preserve data privacy. To this purpose, a combination of stringent protocols (ie, General Data Protection Regulation Compliance; Health Insurance Portability and Accountability Act Guidelines; California Consumer Privacy Act Regulations; ISO 27001 Standards) and advanced technologies must be applied. The key technologies are as follows:

• Data encryption: Data are encrypted both in transit and at rest. This means that any data being transferred over networks or stored in databases are converted into a secure code that can only be decrypted by authorized users. Encryption standards such as advanced encryption standard and Secure Sockets Layer/Transport Layer Security are commonly used to safeguard sensitive information.
• Access controls: Strict access controls are implemented to ensure that only authorized personnel can access sensitive data. This includes multifactor authentication, role-based access control, and regularly updated access permissions. These measures help in limiting data access to only those individuals who need it for their specific role.
• Anonymization and deidentification: To protect personal information, AI tools often anonymize or deidentify data. This process removes or obfuscates personal identifiers, making it difficult to trace the data back to an individual. Techniques include data masking, pseudonymization, and aggregation.
• Data minimization: AI systems are designed to collect only the minimum amount of data necessary for their function. This principle of data minimization reduces the risk of exposure by limiting the volume of sensitive data being handled.
• Secure data storage: Data are stored in secure environments with robust physical and cybersecurity measures. This includes secure servers, data centers with restricted access, and cloud storage solutions that comply with industry standards such as ISO 27001.
• Regular audits and adherence: Regular security audits and adherence checks are conducted to ensure adherence to data protection regulations such as General Data Protection Regulation, Health Insurance Portability and Accountability Act, and California Consumer Privacy Act. These audits help identify and mitigate potential vulnerabilities.
• User consent and transparency: AI tools ensure that users are informed about data collection and use practices through clear privacy policies and consent forms. Users are given control over their data, including options to opt-out of data collection or request data deletion.
• Incident response plans: Robust incident response plans are in place to quickly address any data breaches or security incidents. These plans include protocols for detecting, reporting, and mitigating the impact of data breaches.

Using these advanced privacy-preserving techniques and adhering to strict regulatory standards, AI tools can ensure the confidentiality and security of sensitive data, thereby maintaining user trust and adherence with legal requirements.

## Discussion

This systematic review focused on the transformative impact of AI and CDSA for the diagnosis and treatment of infertility by significantly enhancing personalized medical approaches based on patient predicted outcomes. Table 3 illustrates the predictive capabilities of AI in various stages of ART, from calculating success rates to evaluating embryo quality.

Artificial intelligence technologies use vast data sets to analyze variables such as genetic profiles and lifestyle factors, offering insights for diagnosis and treatment.^4^^,^^135^ Artificial intelligence algorithms excel—when adequately trained on evidence-based pathologies—in identifying subtle variations in medical imaging.^12^^,^^91^ Artificial intelligence technologies are also a valuable tool for treatment decision-making. They assist in optimizing infertility treatment protocols by analyzing patient-specific factors such as BMI and hormone levels, thus personalizing medication dosages and timing.^10^ Moreover, AI enhances embryo assessment by analyzing morphokinetic and genetic data to select embryos with the highest potential for successful pregnancy.^136^ Artificial intelligence’s objective analysis helps in reducing human error in embryo selection, thereby improving the chances of successful implantations.

Artificial intelligence and CDSA have the potential to provide personalized treatment plans that potentially reduce the number of treatment cycles required, lessening the emotional and financial burden on couples. AI is based on learning from the training sets, which is subjective to variability, and that the outcome is simply bound to the different variables examined in the testing process.

Furthermore, AI is expected to improve imaging technologies, integrate genomic data for better predictions, and facilitate remote ART services through telemedicine, enhancing patient accessibility and treatment outcomes. Ethical considerations, including ensuring data privacy and obtaining patient consent, are crucial for responsible AI use in clinical settings.

Artificial intelligence is significantly advancing the field of reproductive medicine by providing more accurate diagnoses and personalized treatment options. Although AI and ML models offer promising advancements, a critical review of their efficacy, limitations, and overall impact is necessary to understand their real-world applicability and potential for improving ART outcomes. In particular, studies evaluating the application of AI should consider the following critical aspects:

• Data quality and quantity: The effectiveness of AI/ML models depends heavily on the quality and quantity of data available. Inconsistent or incomplete data can lead to inaccurate predictions.
• Generalizability: Models trained on specific data sets may not perform well when applied to different populations or clinical settings. This raises concerns about the generalizability of the results.
• Interpretability: Many AI/ML models, particularly deep learning models, operate as “black boxes,” making it difficult to understand how decisions are made. This lack of transparency can be a barrier to clinical acceptance and patient trust.
• Ethical and legal concerns: The use of patient data in AI/ML models raises ethical and legal questions about privacy, consent, and data security. Depending on specific country body laws different countries may interpretate the AI/ML integration in the clinical practice as issue to persecute or value to encourage. However, the use of AI/ML remains the doctor’s legal responsibility. In fact, in several countries, it is not possible to transfer that responsibility from doctor to AI/ML tools.

Notably, the Federal Drug Administration now regulates CDSA systems as medical devices, emphasizing the importance of reliable predictions in clinical decision-making.^5^ The use of AI and CDSA will not replace physicians and embryologists, but it will help enhance efficiency and quality of work, thereby increasing access to care, reducing costs, and waiting times.^137^

## Conclusion

In conclusion, the advent of automated technologies is transforming the field of infertility diagnosis and treatment. Algorithms have the potential to expedite and enhance the accuracy of diagnosis, guide personalized treatment, and improve overall patient care. As in some specific medical applications, AI continues to evolve.^138^^,^^139^ In the near future, we can expect further advances that will bring new hope to couples struggling with infertility, to help achieve their dream of starting a family.^140^ Indeed, automated tools may enable researchers and clinicians to gain deeper insights into the complex dynamics of fertility. By integrating diverse data sets and simulating various scenarios, these models promise to enhance diagnostic penetrance, optimize treatment strategies, and improve treatment outcomes for infertile couples.

Overall, the validated use of AI, mathematical models, algorithms, and calculators in the clinical management of infertile couples during OS yields significant advantages. These tools prove invaluable not only in subsequent steps of IVF procedures but also during laboratory/embryology management and embryo transfer. However, further research and validation studies are essential to continually refine and broaden the applications of these tools in both clinical and laboratory settings. Indeed, at the current stage, it is not possible to draw any conclusions from the results of this review owing to the lack of standardization in validation methods across studies. Further validation and clinical trials are needed to establish the effectiveness of these tools in the clinical setting.

With ongoing advancements, the integration of AI, mathematical models, algorithms, and calculators promises to further enhance the precision and efficacy of medical and embryologic protocols, thereby leading to improved treatment outcomes and heightened success rates in ART. In the foreseeable future, a superalgorithm that integrates all computer and AI tools that have been clinically validated could be adopted in the ART clinics by fertility experts and embryologists, leading to significant benefits for all patients involved.

## Potential Competing Interests

The authors report no competing interests.

## Declaration of Generative AI and AI-assisted Technologies in the Writing Process

The authors employed AI and AI-assisted technologies to enhance the readability and language of this work. These tools were used to support, not replace, essential authoring tasks such as generating scientific, pedagogic, or medical insights, drawing scientific conclusions, and providing clinical recommendations. Human oversight and control were maintained throughout the writing process, and all work was carefully reviewed and edited. The authors remain fully responsible and accountable for the content of this work.

## References

[1] European IVF Monitoring Consortium (EIM) for the European Society of Human Reproduction and Embryology (ESHRE), Smeenk J., Wyns C., Geyter De (2023). ART in Europe, 2019: results generated from European registries by ESHRE. Hum Reprod.

[2] Aimagambetova G., Issanov A., Terzic S. (2020). The effect of psychological distress on IVF outcomes: reality or speculations?. PLoS One.

[3] Ben Rafael Z. (2020). Repeated implantation failure (RIF): an iatrogenic meaningless definition that generates unnecessary and costly use of add-on procedures. Hum Reprod.

[4] Hanassab S., Abbara A., Yeung A.C. (2024). The prospect of artificial intelligence to personalize assisted reproductive technology. NPJ Digit Med.

[5] Goodman K.E., Rodman A.M., Morgan D.J. (2023). Preparing physicians for the clinical algorithm era. N Engl J Med.

[6] McLernon D.J., Raja E.-A., Toner J.P. (2022). Predicting personalized cumulative live birth following in vitro fertilization. Fertil Steril.

[7] Thirunavukarasu A.J., Ting D.S.J., Elangovan K., Gutierrez L., Tan T.F., Ting D.S.W. (2023). Large language models in medicine. Nat Med.

[8] Fanton M., Nutting V., Solano F. (2022). An interpretable machine learning model for predicting the optimal day of trigger during ovarian stimulation. Fertil Steril.

[9] Fanton M., Nutting V., Rothman A. (2022). An interpretable machine learning model for individualized gonadotrophin starting dose selection during ovarian stimulation. Reprod Biomed Online.

[10] Wang C.-W., Kuo C.-Y., Chen C.-H., Hsieh Y.-H., Su E.C.-Y. (2022). Predicting clinical pregnancy using clinical features and machine learning algorithms in in vitro fertilization. PLoS One.

[11] Moher D., Liberati A., Tetzlaff J., Altman D.G., PRISMA Group (2009). Preferred reporting items for systematic reviews and meta-analyses: the PRISMA statement. PLoS Med.

[12] Chapron C., Lafay-Pillet M.-C., Santulli P. (2022). A new validated screening method for endometriosis diagnosis based on patient questionnaires. EclinicalMedicine.

[13] Esteves S.C., Roque M., Sunkara S.K. (2019). Oocyte quantity, as well as oocyte quality, plays a significant role for the cumulative live birth rate of a POSEIDON criteria patient. Hum Reprod.

[14] Xu H., Feng G., Shi L., Han Y., Huang Q., Li R. (2023). PCOSt: A non-invasive and cost-effective screening tool for polycystic ovary syndrome. Innovation (Camb).

[15] Correia K.F.B., Missmer S.A., Weinerman R., Ginsburg E.S., Rossi B.V. (2023). Development of a model to estimate the optimal number of oocytes to attempt to fertilize during assisted reproductive technology treatment. JAMA Netw Open.

[16] Ata B., Kalafat E., Somigliana E. (2021). A new definition of recurrent implantation failure on the basis of anticipated blastocyst aneuploidy rates across female age. Fertil Steril.

[17] Zad Z., Jiang V.S., Wolf A.T. (2024). Predicting polycystic ovary syndrome with machine learning algorithms from electronic health records. Front Endocrinol (Lausanne).

[18] Kaufmann S.J., Eastaugh J.L., Snowden S., Smye S.W., Sharma V. (1997). The application of neural networks in predicting the outcome of in- vitro fertilization. Hum Reprod.

[19] Wald M., Sparks A.E.T., Sandlow J., Van-Voorhis B., Syrop C.H., Niederberger C.S. (2005). Computational models for prediction of IVF/ICSI outcomes with surgically retrieved spermatozoa. Reprod Biomed Online.

[20] Jones C.A., Christensen A.L., Salihu H. (2011). Prediction of individual probabilities of livebirth and multiple birth events following in vitro fertilization (IVF): a new outcomes counselling tool for IVF providers and patients using HFEA metrics. J Exp Clin Assist Reprod.

[21] Uyar A., Bener A., Ciray H.N. (2015). Predictive modeling of implantation outcome in an in vitro fertilization setting: an application of machine learning methods. Med Decis Making.

[22] Milewski R., Kuczyńska A., Stankiewicz B., Kuczyński W. (2017). How much information about embryo implantation potential is included in morphokinetic data? A prediction model based on artificial neural networks and principal component analysis. Adv Med Sci.

[23] Burai P., Hajdu A., Manuel F.E., Harangi B. (2018). Segmentation of the uterine wall by an ensemble of fully convolutional neural networks. Annu Int Conf IEEE Eng Med Biol Soc.

[24] Leijdekkers J.A., Eijkemans M.J.C., van Tilborg T.C. (2018). Predicting the cumulative chance of live birth over multiple complete cycles of in vitro fertilization: an external validation study. Hum Reprod.

[25] Blank C., Wildeboer R.R., DeCroo I. (2019). Prediction of implantation after blastocyst transfer in in vitro fertilization: a machine-learning perspective. Fertil Steril.

[26] Qiu J., Li P., Dong M., Xin X., Tan J. (2019). Personalized prediction of live birth prior to the first in vitro fertilization treatment: a machine learning method. J Transl Med.

[27] Vogiatzi P., Pouliakis A., Siristatidis C. (2019). An artificial neural network for the prediction of assisted reproduction outcome. J Assist Reprod Genet.

[28] Xu B., Liu C., Qian L. (2019). Statistical modelling outcome of in vitro fertilization and intracytoplasmic sperm injection: a single centre study. Comb Chem High Throughput Screen.

[29] Barnett-Itzhaki Z., Elbaz M., Butterman R. (2020). Machine learning vs. classic statistics for the prediction of IVF outcomes. J Assist Reprod Genet.

[30] Ferrick L., Lee Y.S.L., Gardner D.K. (2020). Metabolic activity of human blastocysts correlates with their morphokinetics, morphological grade, KIDScore and artificial intelligence ranking. Hum Reprod.

[31] Goyal A., Kuchana M., Ayyagari K.P.R. (2020). Machine learning predicts live-birth occurrence before in-vitro fertilization treatment. Sci Rep.

[32] Letterie G., MacDonald A. (2020). Artificial intelligence in in vitro fertilization: a computer decision support system for day-to-day management of ovarian stimulation during in vitro fertilization. Fertil Steril.

[33] Liao S., Pan W., Dai W.-Q. (2020). Development of a dynamic diagnosis grading system for infertility using machine learning. JAMA Netw Open.

[34] Liu L., Jiao Y., Li X., Ouyang Y., Shi D. (2020). Machine learning algorithms to predict early pregnancy loss after in vitro fertilization-embryo transfer with fetal heart rate as a strong predictor. Comput Methods Programs Biomed.

[35] Liu R., Bai S., Jiang X. (2021). Multifactor prediction of embryo transfer outcomes based on a machine learning algorithm. Front Endocrinol (Lausanne).

[36] Liu H., Zhang Z., Gu Y. (2023). Development and evaluation of a live birth prediction model for evaluating human blastocysts from a retrospective study. Elife.

[37] Raef B., Maleki M., Ferdousi R. (2020). Computational prediction of implantation outcome after embryo transfer. Health Informatics J.

[38] Tarín J.J., Pascual E., García-Pérez M.A., Gómez R., Hidalgo-Mora J.J., Cano A. (2020). A predictive model for women’s assisted fecundity before starting the first IVF/ICSI treatment cycle. J Assist Reprod Genet.

[39] Huang B., Tan W., Li Z., Jin L. (2021). An artificial intelligence model (euploid prediction algorithm) can predict embryo ploidy status based on time-lapse data. Reprod Biol Endocrinol.

[40] Jin H., Shen X., Song W., Liu Y., Qi L., Zhang F. (2021). The development of nomograms to predict blastulation rate following cycles of in vitro fertilization in patients with tubal factor infertility, polycystic ovary syndrome, or endometriosis. Front Endocrinol (Lausanne).

[41] Kozar N., Kovač V., Reljič M. (2021). Can methods of artificial intelligence aid in optimizing patient selection in patients undergoing intrauterine inseminations?. J Assist Reprod Genet.

[42] Li F., Lu R., Zeng C., Li X., Xue Q. (2021). Development and validation of a clinical pregnancy failure prediction model for poor ovarian responders during IVF/ICSI. Front Endocrinol (Lausanne).

[43] Srivastava D., Gupta S., Kudavelly S., Suryanarayana K.V., Ga R. (2021). Unsupervised deep learning based longitudinal follicular growth tracking during IVF cycle using 3D transvaginal ultrasound in assisted reproduction. Annu Int Conf IEEE Eng Med Biol Soc.

[44] Bardet L., Excoffier J.-B., Salaun-Penquer N. (2022). Comparison of predictive models for cumulative live birth rate after treatment with ART. Reprod Biomed Online.

[45] Fu K., Li Y., Lv H., Wu W., Song J., Xu J. (2022). Development of a model predicting the outcome of in vitro fertilization cycles by a robust decision tree method. Front Endocrinol.

[46] Kuroda S., Karna K.K., Kaiyal R.S., Gupta S., Sharma R., Agarwal A. (2022). Development of a novel robust artificial intelligence developed sperm DNA fragmentation test—preliminary findings. Fertil Steril.

[47] Mehrjerd A., Rezaei H., Eslami S., Ratna M.B., Khadem Ghaebi N. (2022). Internal validation and comparison of predictive models to determine success rate of infertility treatments: a retrospective study of 2485 cycles. Sci Rep.

[48] Mehrjerd A., Rezaei H., Eslami S., Khadem Ghaebi N. (2022). Determination of cut off for endometrial thickness in couples with unexplained infertility: trustable AI. Stud Health Technol Inform.

[49] Mercuri N., Fjeldstad J., Krivoi A., Meriano J., Nayot D. (2022). A non-invasive, 2-dimensional (2D) image analysis artificial intelligence (AI) tool scores mature oocytes and correlates with the quality of subsequent blastocyst development. Fertil Steril.

[50] Qu P., Chen L., Zhao D., Shi W., Shi J. (2022). Nomogram for the cumulative live birth in women undergoing the first IVF cycle: Base on 26, 689 patients in China. Front Endocrinol (Lausanne).

[51] Shen L., Zhang Y., Chen W., Yin X. (2022). The application of artificial intelligence in predicting embryo transfer outcome of recurrent implantation failure. Front Physiol.

[52] Wen J.-Y., Liu C.-F., Chung M.-T., Tsai Y.-C. (2022). Artificial intelligence model to predict pregnancy and multiple pregnancy risk following in vitro fertilization-embryo transfer (IVF-ET). Taiwan J Obstet Gynecol.

[53] Yang L., Peavey M., Kaskar K. (2022). Development of a dynamic machine learning algorithm to predict clinical pregnancy and live birth rate with embryo morphokinetics. F S Rep.

[54] Cheredath A., Uppangala S., Asha C.S. (2023). Combining machine learning with metabolomic and embryologic data improves embryo implantation prediction. Reprod Sci.

[55] Gong X., Zhang Y., Zhu Y. (2023). Development and validation of a live birth prediction model for expected poor ovarian response patients during IVF/ICSI. Front Endocrinol (Lausanne).

[56] Louis C.M., Handayani N., Aprilliana T., Polim A.A., Boediono A., Sini I. (2023). Genetic algorithm-assisted machine learning for clinical pregnancy prediction in in vitro fertilization. AJOG Glob Rep.

[57] Ozer G., Akca A., Yuksel B., Duzguner I., Pehlivanli A.C., Kahraman S. (2023). Prediction of risk factors for first trimester pregnancy loss in frozen-thawed good-quality embryo transfer cycles using machine learning algorithms. J Assist Reprod Genet.

[58] Yuan G., Lv B., Du X. (2023). Prediction model for missed abortion of patients treated with IVF-ET based on XGBoost: a retrospective study. PeerJ.

[59] Canosa S., Licheri N., Bergandi L. (2024). A novel machine-learning framework based on early embryo morphokinetics identifies a feature signature associated with blastocyst development. J Ovarian Res.

[60] Yi W.J., Park K.S., Paick J.S. (1998). Morphological classification of sperm heads using artificial neural networks. Stud Health Technol Inform.

[61] Valiuškaitė V., Raudonis V., Maskeliūnas R., Damaševičius R., Krilavičius T. (2020). Deep learning based evaluation of spermatozoid motility for artificial insemination. Sensors (Basel).

[62] Javadi S., Mirroshandel S.A. (2019). A novel deep learning method for automatic assessment of human sperm images. Comput Biol Med.

[63] McCallum C., Riordon J., Wang Y. (2019). Deep learning-based selection of human sperm with high DNA integrity. Commun Biol.

[64] Abbasi A., Miahi E., Mirroshandel S.A. (2021). Effect of deep transfer and multi-task learning on sperm abnormality detection. Comput Biol Med.

[65] Jiang A., Jiaqi W., Zhao H., Zhang Z., Sun Y. (2022). Identifying viability of immotile sperm at one glance: sperm viability classifier powered by deep learning. Fertil Steril.

[66] Mendizabal-Ruiz G., Chavez-Badiola A., Aguilar Figueroa I. (2022). Computer software (SiD) assisted real-time single sperm selection associated with fertilization and blastocyst formation. Reprod Biomed Online.

[67] Ottl S., Amiriparian S., Gerczuk M., Schuller B.W. (2022). motilitAI: a machine learning framework for automatic prediction of human sperm motility. iScience.

[68] Saiffe Farías A.F., Sakkas D., Chavez-Badiola A. (2022). Single-sperm motility analysis during icsi using an artificial intelligence sperm identification software (SID) and correlation with morphology. Fertil Steril.

[69] Peng T., Liao C., Ye X. (2023). Machine learning-based clustering to identify the combined effect of the DNA fragmentation index and conventional semen parameters on in vitro fertilization outcomes. Reprod Biol Endocrinol.

[70] Kobanawa M. (2023). The gonadotropins starting dose calculator, which can be adjusted the target number of oocytes and stimulation duration days to achieve individualized controlled ovarian stimulation in Japanese patients. Reprod Med Biol.

[71] Howles C.M., Saunders H., Alam V., Engrand P., FSH Treatment Guidelines Clinical Panel (2006). Predictive factors and a corresponding treatment algorithm for controlled ovarian stimulation in patients treated with recombinant human follicle stimulating hormone (follitropin alfa) during assisted reproduction technology (ART) procedures. An analysis of 1378 patients. Curr Med Res Opin.

[72] Olivennes F., Trew G., Borini A. (2015). Randomized, controlled, open-label, non-inferiority study of the CONSORT algorithm for individualized dosing of follitropin alfa. Reprod Biomed Online.

[73] Hariton E., Chi E.A., Chi G. (2021). A machine learning algorithm can optimize the day of trigger to improve in vitro fertilization outcomes. Fertil Steril.

[74] Correa N., Cerquides J., Arcos J.L., Vassena R. (2022). Supporting first FSH dosage for ovarian stimulation with machine learning. Reprod Biomed Online.

[75] Letterie G., MacDonald A., Shi Z. (2022). An artificial intelligence platform to optimize workflow during ovarian stimulation and IVF: process improvement and outcome-based predictions. Reprod Biomed Online.

[76] Morales D.A., Bengoetxea E., Larrañaga P. (2008). Bayesian classification for the selection of in vitro human embryos using morphological and clinical data. Comput Methods Programs Biomed.

[77] Manna C., Nanni L., Lumini A., Pappalardo S. (2013). Artificial intelligence techniques for embryo and oocyte classification. Reprod Biomed Online.

[78] VerMilyea M.D., Tan L., Anthony J.T. (2014). Computer-automated time-lapse analysis results correlate with embryo implantation and clinical pregnancy: a blinded, multi-centre study. Reprod Biomed Online.

[79] VerMilyea M., Hall J.M.M., Diakiw S.M. (2020). Development of an artificial intelligence-based assessment model for prediction of embryo viability using static images captured by optical light microscopy during IVF. Hum Reprod.

[80] Diamond M.P., Suraj V., Behnke E.J. (2015). Using the Eeva Test^TM^ adjunctively to traditional day 3 morphology is informative for consistent embryo assessment within a panel of embryologists with diverse experience. J Assist Reprod Genet.

[81] Petersen B.M., Boel M., Montag M., Gardner D.K. (2016). Development of a generally applicable morphokinetic algorithm capable of predicting the implantation potential of embryos transferred on Day 3. Hum Reprod.

[82] Adolfsson E., Porath S., Andershed A.N. (2018). External validation of a time-lapse model: a retrospective study comparing embryo evaluation using a morphokinetic model to standard morphology with live birth as endpoint. JBRA Assist Reprod.

[83] Bodri D., Milewski R., Yao Serna J. (2018). Predicting live birth by combining cleavage and blastocyst-stage time-lapse variables using a hierarchical and a data mining-based statistical model. Reprod Biol.

[84] Rocafort E., Enciso M., Leza A., Sarasa J., Aizpurua J. (2018). Euploid embryos selected by an automated time-lapse system have superior SET outcomes than selected solely by conventional morphology assessment. J Assist Reprod Genet.

[85] Storr A., Venetis C., Cooke S., Kilani S., Ledger W. (2018). Time-lapse algorithms and morphological selection of day-5 embryos for transfer: a preclinical validation study. Fertil Steril.

[86] Strouthopoulos C., Anifandis G. (2018). An automated blastomere identification method for the evaluation of day 2 embryos during IVF/ICSI treatments. Comput Methods Programs Biomed.

[87] Alegre L., Del Gallego R., Arrones S., Hernández P., Muñoz M., Meseguer M. (2019). Novel noninvasive embryo selection algorithm combining time-lapse morphokinetics and oxidative status of the spent embryo culture medium. Fertil Steril.

[88] Dirvanauskas D., Maskeliunas R., Raudonis V., Damasevicius R. (2019). Embryo development stage prediction algorithm for automated time lapse incubators. Comput Methods Programs Biomed.

[89] Kanakasabapathy M.K., Thirumalaraju P., Bormann C.L. (2019). Development and evaluation of inexpensive automated deep learning-based imaging systems for embryology. Lab Chip.

[90] Khosravi P., Kazemi E., Zhan Q. (2019). Deep learning enables robust assessment and selection of human blastocysts after in vitro fertilization. NPJ Digit Med.

[91] Kovacs P., Matyas S., Forgacs V., Sajgo A., Molnar L., Pribenszky C. (2019). Non-invasive embryo evaluation and selection using time-lapse monitoring: results of a randomized controlled study. Eur J Obstet Gynecol Reprod Biol.

[92] Kragh M.F., Rimestad J., Berntsen J., Karstoft H. (2019). Automatic grading of human blastocysts from time-lapse imaging. Comput Biol Med.

[93] Liu Y., Feenan K., Chapple V., Matson P. (2019). Assessing efficacy of day 3 embryo time-lapse algorithms retrospectively: impacts of dataset type and confounding factors. Hum Fertil (Camb).

[94] Reignier A., Girard J.-M., Lammers J. (2019). Performance of Day 5 KIDScore^TM^ morphokinetic prediction models of implantation and live birth after single blastocyst transfer. J Assist Reprod Genet.

[95] Tran D., Cooke S., Illingworth P.J., Gardner D.K. (2019). Deep learning as a predictive tool for fetal heart pregnancy following time-lapse incubation and blastocyst transfer. Hum Reprod.

[96] Bori L., Paya E., Alegre L. (2020). Novel and conventional embryo parameters as input data for artificial neural networks: an artificial intelligence model applied for prediction of the implantation potential. Fertil Steril.

[97] Chavez-Badiola A., Flores-Saiffe-Farías A., Mendizabal-Ruiz G., Drakeley A.J., Cohen J. (2020). Embryo Ranking Intelligent Classification Algorithm (ERICA): artificial intelligence clinical assistant predicting embryo ploidy and implantation. Reprod Biomed Online.

[98] Feyeux M., Reignier A., Mocaer M. (2020). Development of automated annotation software for human embryo morphokinetics. Hum Reprod.

[99] Fishel S., Campbell A., Foad F. (2020). Evolution of embryo selection for IVF from subjective morphology assessment to objective time-lapse algorithms improves chance of live birth. Reprod Biomed Online.

[100] Fukunaga N., Sanami S., Kitasaka H. (2020). Development of an automated two pronuclei detection system on time-lapse embryo images using deep learning techniques. Reprod Med Biol.

[101] Blais I., Koifman M., Feferkorn I., Dirnfeld M., Lahav-Baratz S. (2021). Improving embryo selection by the development of a laboratory-adapted time-lapse model. F S Sci.

[102] Coticchio G., Fiorentino G., Nicora G. (2021). Cytoplasmic movements of the early human embryo: imaging and artificial intelligence to predict blastocyst development. Reprod Biomed Online.

[103] Fitz V.W., Kanakasabapathy M.K., Thirumalaraju P. (2021). Should there be an ‘AI’ in TEAM? Embryologists selection of high implantation potential embryos improves with the aid of an artificial intelligence algorithm. J Assist Reprod Genet.

[104] Friedenthal J., Hernandez-Nieto C., Roth R.M. (2021). Clinical implementation of algorithm-based embryo selection is associated with improved pregnancy outcomes in single vitrified warmed euploid embryo transfers. J Assist Reprod Genet.

[105] Geller J., Collazo I., Pai R. (2021). An artificial intelligence-based algorithm for predicting pregnancy success using static images captured by optical light microscopy during intracytoplasmic sperm injection. J Hum Reprod Sci.

[106] Giscard d’Estaing S., Labrune E., Forcellini M. (2021). A machine learning system with reinforcement capacity for predicting the fate of an ART embryo. Syst Biol Reprod Med.

[107] Lee C.-I., Su Y.-R., Chen C.-H. (2021). End-to-end deep learning for recognition of ploidy status using time-lapse videos. J Assist Reprod Genet.

[108] Liao Q., Zhang Q., Feng X. (2021). Development of deep learning algorithms for predicting blastocyst formation and quality by time-lapse monitoring. Commun Biol.

[109] Sawada Y., Sato T., Nagaya M. (2021). Evaluation of artificial intelligence using time-lapse images of IVF embryos to predict live birth. Reprod Biomed Online.

[110] Targosz A., Przystałka P., Wiaderkiewicz R., Mrugacz G. (2021). Semantic segmentation of human oocyte images using deep neural networks. Biomed Eng Online.

[111] Thirumalaraju P., Kanakasabapathy M.K., Bormann C.L. (2021). Evaluation of deep convolutional neural networks in classifying human embryo images based on their morphological quality. Heliyon.

[112] Ueno S., Berntsen J., Ito M. (2021). Pregnancy prediction performance of an annotation-free embryo scoring system on the basis of deep learning after single vitrified-warmed blastocyst transfer: a single-center large cohort retrospective study. Fertil Steril.

[113] Xi Q., Yang Q., Wang M. (2021). Individualized embryo selection strategy developed by stacking machine learning model for better in vitro fertilization outcomes: an application study. Reprod Biol Endocrinol.

[114] Zhao M., Xu M., Li H. (2021). Application of convolutional neural network on early human embryo segmentation during in vitro fertilization. J Cellular Molecular Med.

[115] Ahlström A., Lundin K., Lind A.-K. (2022). A double-blind randomized controlled trial investigating a time-lapse algorithm for selecting Day 5 blastocysts for transfer. Hum Reprod.

[116] Berntsen J., Rimestad J., Lassen J.T., Tran D., Kragh M.F. (2022). Robust and generalizable embryo selection based on artificial intelligence and time-lapse image sequences. PLoS One.

[117] Diakiw S.M., Hall J.M.M., VerMilyea M. (2022). An artificial intelligence model correlated with morphological and genetic features of blastocyst quality improves ranking of viable embryos. Reprod Biomed Online.

[118] Enatsu N., Miyatsuka I., An L.M. (2022). A novel system based on artificial intelligence for predicting blastocyst viability and visualizing the explanation. Reprod Med Biol.

[119] Bamford T., Easter C., Montgomery S. (2023). A comparison of 12 machine learning models developed to predict ploidy, using a morphokinetic meta-dataset of 8147 embryos. Hum Reprod.

[120] Barnes J., Brendel M., Gao V.R. (2023). A non-invasive artificial intelligence approach for the prediction of human blastocyst ploidy: a retrospective model development and validation study. Lancet Digit Health.

[121] Ferrand T., Boulant J., He C. (2023). Predicting the number of oocytes retrieved from controlled ovarian hyperstimulation with machine learning. Hum Reprod.

[122] Herbert S.-L., Staib C., Wallner T. (2023). Morphokinetic analysis of early human embryonic development and its relationship to endometriosis resection: a retrospective time-lapse study using the KIDScore^TM^ D3 and D5 implantation data algorithm. Arch Gynecol Obstet.

[123] Zieliński K., Pukszta S., Mickiewicz M. (2023). Personalized prediction of the secondary oocytes number after ovarian stimulation: A machine learning model based on clinical and genetic data. PLoS Comput Biol.

[124] Ferraretti A.P., La Marca A., Fauser B.C. (2011). ESHRE consensus on the definition of ‘poor response’ to ovarian stimulation for in vitro fertilization: the Bologna criteria. Hum Reprod.

[125] Cherouveim P., Velmahos C., Bormann C.L. (2023). Artificial intelligence for sperm selection-a systematic review. Fertil Steril.

[126] Andrabi S.W., Ara A., Saharan A., Jaffar M., Gugnani N., Esteves S.C. (2024). Sperm DNA fragmentation: causes, evaluation and management in male infertility. JBRA Assist Reprod.

[127] Bulletti F.M., Berrettini M., De Luca R. (2023). Calculators of predictive results: empowering assisted reproductive technologies programs. Clin Med.

[128] Sinai I., Igras S., Lundgren R. (2017). A practical alternative to calculating unmet need for family planning. Open Access J Contracept.

[129] (April 26, 2023). *Infertility*.

[130] Huang J., Lv P., Lian Y. (2022). Construction of machine learning tools to predict threatened miscarriage in the first trimester based on AEA, progesterone and β-hCG in China: a multicentre, observational, case-control study. BMC Pregnancy Childbirth.

[131] Guo X., Zhan H., Zhang X. (2023). Predictive models for starting dose of gonadotropin in controlled ovarian hyperstimulation: review and progress update. Hum Fertil (Camb).

[132] Kragh M.F., Karstoft H. (2021). Embryo selection with artificial intelligence: how to evaluate and compare methods?. J Assist Reprod Genet.

[133] Kirkegaard K., Agerholm I.E., Ingerslev H.J. (2012). Time-lapse monitoring as a tool for clinical embryo assessment. Hum Reprod.

[134] Kirkegaard K., Ahlström A., Ingerslev H.J., Hardarson T. (2015). Choosing the best embryo by time lapse versus standard morphology. Fertil Steril.

[135] Leung E.T.Y., Lee C.-L., Tian X. (2022). Simulating nature in sperm selection for assisted reproduction. Nat Rev Urol.

[136] Afnan M.A.M., Liu Y., Conitzer V. (2021). Interpretable, not black-box, artificial intelligence should be used for embryo selection. Hum Reprod Open.

[137] Costa-Borges N., Munné S., Albó E. (2023). First babies conceived with automated intracytoplasmic sperm injection. Reprod Biomed Online.

[138] Lehman C.D., Wellman R.D., Buist D.S.M. (2015). Diagnostic accuracy of igital screening mammography with and without computer-aided detection. JAMA Intern Med.

[139] FDA (March 15, 2024). Artificial intelligence and machine learning in software as a medical device. FDA.

[140] Habbema J.D.F., Eijkemans M.J.C., Leridon H., te Velde E.R. (2015). Realizing a desired family size: when should couples start?. Hum Reprod.

