# Supplementary material for: Artificial Intelligence, Clinical Decision Support Algorithms, Mathematical Models, Calculators Applications in Infertility: Systematic Review and Hands-On Digital Applications
Source: Mayo Clin Proc Digit Health. 2024 Aug 26;2(4):518–32. doi: 10.1016/j.mcpdig.2024.08.007 (PMC11975849; doi:10.1016/j.mcpdig.2024.08.007)
Supplement: Supplementary Table 1 [file mmc1.pdf]

Embryo quality

REFERENCES	STUDY DESIGN	TECHNIQUE	POPULATION	SAMPLE SIZE	INTERVENTION	COMPARISON	OUTCOME VARIABLES	RESULTS	MEAN DIFFERENCE	P
(Herbert et al., 2023)	Retrospective study	Embryo selection	128 patients	237 fertilized, cultured and transferred embryos	Time-lapse microscopy used to assess embryo quality after complete resection of endometriosis	Time-lapse microscopy used to assess embryo quality without complete resection of endometriosis	Impact of resection on embryo quality	KIDScore™ D3: 3 vs 4<br>KIDScore™ D5: 7.2 vs 2.6		0.668<br>0.002
							Pregnancy rate	32.6% vs 17.6%		0.346
							Abortion rate	44.4% vs 100%		0.455
(Enatsu et al., 2022)	Retrospective study	Embryo selection	9,961 patients	19,342 blastocyst images (training set: 17,984; testing set: 1,358)	AI system Fertility image Testing Through Embryo (FiTTE)	Conventional Gardner scoring system	Predict clinical pregnancy using blastocyst images and clinical data	Prediction accuracy: 65.2 vs 59.8%<br>AUC: 0.71 vs 0.62		<0.01<br><br><0.01
(Ahlström et al., 2022)	RCT	Fresh single embryo transfer	Patients with at least two good quality blastocysts on Day 5	776 patients (387 patients in the group)	Time-lapse blastocyst selection model	Human selection	Primary: Ongoing pregnancy rate (foetal heart activity at 6–8 weeks).	47.4% vs 48.1%	−0.7% (95% CI: −8.2, 6.7)	0.90
							Secondary: Pregnancy (a positive beta-hCG urinary test 16 days after embryo transfer)	60.2% vs 59.0%,	1.1% (95% CI: −6.2, 8.4)	0.81
							Early pregnancy loss	21.2% vs 18.5%	2.7% (95% CI: −5.2, 10.6)	0.55
							Agreement (Y/N) between the preliminary decisions based on morphology after the algorithm was applied	No: 42% of occasions after the KIDScore™ D5	95% CI: 36.9, 47.2	
(Berntsen et al., 2022)	Retrospective study	Embryo selection	Data from 18 clinics worldwide from between 2011 and 2019	115,832-time lapse images (14,644: KID embryos; training data set: 85%; independent data set: 15%)	Automated deep learning model (iDAScore v1.0)	Manual morphokinetic model (KIDScore D5 v3 model)	Sort FH-/FH+ embryos	AUC<br>For sorting all embryos:<br>for iDAScore	0.92 vs 0.89<br>(95% CI: 0.95–0.96)	
								For sorting KID embryos:<br>for IDAScore	0.67 vs 0.66<br>(95% CI: 0.64-0.69)	
(Sawada et al., 2021)	Retrospective study	Embryo selection	175 patients	141,444-time lapse images of 470 transferred embryos (91: live birth; 379: non-live birth)	AI system	Conventional morphological evaluation	Predict the probability of live births	Sensitivity	0.581 vs 0.603	
								Specificity	0.673 vs 0.617	
								Positive/negative predictive values of the confidence score	0.303/0.872 vs 0.257/0.866	
								Rate of concordance (Cohen kappa coefficient)	–0.049 (95% CI: –0.154-0.056)	
(Fitz et al., 2021)	Retrospective study	Embryo selection	160 patients from 2014 to 2018 at Massachusetts	200 sets of time-lapse images of 400 day 5 euploid embryos	Decision of 14 highly trained embryologists with	Decision of 14 highly trained embryologists without the aid of a	Implantation	Improvement of successfully implanted embryo selection	73.6% (SD 5%) vs 65.5% (SD 5.2%)	

			General Hospital Fertility Center		the aid of a deep learning algorithm	deep learning algorithm		Mean percent improvement	11.1% (range 1.4% to 15.5%)	
(Ueno et al., 2021)	Retrospective study	Embryo selection	3,018 patients from 2019 to 2020	3,018 patients divided in 5 maternal age groups:<br><35 years age group (n=389); 35–37 years age group (n=514); 38–40 years age group (n=796); 41–42 years age group (n=636); >42 years age group (n=389)	iDAScore v1.0 (automatic embryo scoring system)	KIDScore D5 v3 (annotation-dependent morphokinetic embryo scoring model) and the Gardner criteria (traditional morphological grading model)	Predict fetal heartbeat status (FHB) for each maternal group.	AUC<br>*<35 years age group*<br>iDAScore: 0.72<br>KIDScore: 0.64<br>Gardner criteria: 0.64<br><br>*35–37 years age group*:<br>iDAScore: 0.68<br>KIDScore: 0.68<br>Gardner criteria: 0.65<br><br>*38–40 years age* group:<br>iDAScore: 0.67<br>KIDScore: 0.65<br>Gardner criteria: 0.64<br><br>*41–42 years age group:*<br>iDAScore: 0.66<br>KIDScore: 0.66<br>Gardner criteria: 0.63<br><br>*>42 years age group:*<br>iDAScore: 0.76<br>KIDScore: 0.75<br>Gardner criteria: 0.75		
(Targosz et al., 2021)		Oocytes and embryos selection			Comparison of 71 deep neural networks		Semantic oocyte segmentation using deep neural networks	The best score was obtained for one of the variants of DeepLab-v3-ResNet-18 model, when the training accuracy (Acc) reached about 85% for training patterns and 79% for validation ones. The weighted intersection over union (wIoU) and global accuracy (gAcc) for test patterns were calculated, as well. The obtained		

								values of these quality measures were 0,897 and 0.93, respectively.		
(Friedenthal et al., 2021)	Retrospective study	Embryo selection	1,431 patients from 2012 to 2017	924 single vitrified warmed euploid embryo transfers (traditional selection: 1,090; algorithm-based: 3,230)	Mathematical ranking algorithm	Traditional embryo selection via morphologic grading	Implantation rate	65.3% vs 57.8%		<0.0001
							Ongoing pregnancy/live birth rate	54.7% vs 48.1%,		0.0001
							After adjusting for potential confounding variables, utilization of the algorithm remained significantly associated with improved odds of implantation (aOR 1.51, 95% CI: 1.04, 2.18, p=0.03) ongoing pregnancy/live birth (aOR 1.99, 95% CI: 1.38, 2.86, p=0.0002), and decreased odds of clinical loss (aOR 0.42, 95% CI: 0.21, 0.84, p=0.01)			
(Chavez-Badiola et al., 2020)	Retrospective study	Embryo selection	Patients treated between January 2015 and June 2019 at three New Hope Fertility Centers in Mexico City, Guadalajara, and New York City	1,231 blastocyst micrographs	Embryo Ranking Intelligent Classification Algorithm (ERICA)	Random classification and two senior embryologists using normalized discontinued cumulative gain (NDCG)	Euploidy prediction<br><br>An accuracy of 0.70 was obtained with ERICA, with positive predictive value of 0.79 for predicting euploidy and an area under the ROC curve of 0.74 for predicting euploidy. ERICA presents a sensitivity of 0.54 and a specificity of 0.86	ERICA (U=289, p=0.0007) vs embryologist 1 (U=254.5, p=0.0014) vs embryologist 2 (U=246.5, p=0.0242)<br><br>ERICA was tested to see if it was able to find a. The algorithm resulted		
							Detection of euploid blastocyst at the top of the ranking	78.9% (15 out of 19) higher than random classification and the two embryologists		
							Detection of at least one euploid	94.7% (18 out of 19) higher than random		

							embryo within the top two blastocysts	classification and the two embryologists		
(VerMilyea et al., 2020)	Retrospective study	Embryo selection	NA	Standard optical light microscope images of 8,886 Day 5 blastocysts	Life Whisperer AI model	Embryologist’s assessment	Predict embryo vitality	Sensitivity for viable embryos 70.1% specificity for non-viable embryos 60.5% across (3 independent blind test sets from different clinics)		
							Binary comparison of viable/non-viable embryo classification	24.7% higher than embryologists' accuracy (n=2, Student's t test),		0.047
							5-band ranking comparison demonstrated	42.0% higher than embryologists (n=2, Student's t test)		0.028
(Feyeux et al., 2020)	Retrospective study	Embryo selection	NA	701 time-lapse videos (over 60h) (training videos: 78; validation set: 701)	Automated morphokinetic annotation tool (Kinetembryo)	Manual annotation	Morphokinetic parameters annotation accuracy concordance	r2 = 0.92		
(Fishel et al., 2020)	Retrospective study	Embryo selection	NA	1,810 cycles of single blastocyst transfer standard patients (own eggs: 1,373; oocyte donation: 437)	Embryo rank model (time-lapse algorithm, TLIA)	Transfer grade model	Predict the live births outcome	A Vuong non-nested test including covariates showed strong evidence of the superiority of the embryo rank model compared with the transfer grade model (P=0.0008 [raw], P=0.0003 [Akaike information criterion - corrected]). From the receiver operating characteristic (ROC) curves across all possible thresholds the TLIA rank showed better true positive and true negative rates and had a higher area under the curve [AUC] of 67.43% compared with 61.74% for the blastocyst morphology grade.		

								The same analysis but excluding covariates demonstrated an AUC of 62.86% versus 54.02%, respectively.		
(Kragh et al., 2019)	Retrospective study	Embryo selection	NA	Time-lapse images of 8,664 embryos (training set: 80%; validation: 10%; testing: 10%)	Deep learning-based approach	Human assessment	Predict inner cell mass (ICM) and trophectoderm (TE) grades	?		
(Khosravi et al., 2019)	Retrospective study	Embryo selection	NA	12,001 time-lapse images of 10,148 embryos (training group: 70%; 30% validation and test groups)	Deep neural network (DNN) based on Google's Inception-V1 architecture (STORK)	Embryologists classification	Classification of embryo images (good/poor-quality)	STORK predicts blastocyst quality with an AUC of >0.98<br><br>When we applied STORK to these 239 images, we found that it predicted the embryologist majority vote with precision of 95.7% (Cohen's kappa=0.63). In comparison, STORK agreed with each individual embryologist as follows: 0.69, 0.54, 0.25, 0.62, and 0.54 Cohen's kappa score		
(Kovacs et al., 2019)	Randomized controlled study	Embryo quality	NA	161 patients	Time-lapse algorithm	Standard morphological evaluation	Pregnancy rate (primary)	46.3% vs 34.6%	OR: 1.628 (95% CI: 0.857-3.092)	0.150
							Ongoing pregnancy rate (secondary)	42.5% vs 32.1%	OR: 1.54 (95% CI: 1.21-1.96)	0.19
							Pregnancy loss rate	3.8% vs 2.5%	OR: 0.66 (95% CI: 0.46-0.93)	
							Live birth rate	42.5% vs 32.1%	OR: 1.66 (95% CI: 1.13-2.45)	0.19
(Bodri et al., 2018)	Retrospective study	Embryo quality	NA	285 single vitrified-thawed blastocyst transfers	Hierarchical model algorithm	Data-mining model algorithm	Live birth rate prediction	AUC: 0.723 (95% CI: 0.66-0.79) vs 0.717 (95% CI: 0.65-0.78)		
(Rocafort et al., 2018)	Retrospective study	Embryo quality	244 patients between the ages of 19-34 years, had normal menstrual cycles (26–34 days), (101:	Embryo time lapse images	Automated TLI system (Eeva) (PGS-TLI group)	Morphology evaluation alone (PGS-only group)	Implantation rate			
							Clinical pregnancy rate			
							Ongoing pregnancy rate			

			PGS-only group; 143: PGS-TLI group)							
(Storr et al., 2018)	Prospective study	Embryo selection	NA	428 day-5 embryos	Time-lapse algorithms (Algorithm A: Meseguer et al., 2011; algorithm B: Conaghan et al., 2013; algorithm C: VerMilyea et al., 2014; algorithm D: Basile et al., 2015; algorithm E: Goodman et al., 2016; algorithm F: Liu et al., 2016; algorithm G: Petersen et al., 2016)	Ten embryologists using conventional morphologic criteria	Interalgorithm agreement in the selection of the best day-5 embryo for transfer<br><br>Agreement between algorithms and the majority of embryologist in selecting the best day-5 embryo for transfer.<br><br>Kappa score: <0.20 poor; 0.21-0.40 fair; 0.41-0.60 moderate; 0.61-0.80 good; 0.81-1 very good	BEST CASE SCENARIO<br>*Algorithm A*<br>*Agreement*: 48.9 (95% CI: 38.8–59.0)<br>kappa score: 0.337 (95% CI: 0.204–0.456)<br>*Algorithm B*<br>*Agreement*: 72.2 (95% CI: 62.2–80.4)<br>kappa score: 0.629 95% CI (0.502–758)<br>*Algorithm C*<br>*Agreement*: 62.2 (95% CI: 51.9–71.5)<br>kappa score: 0.494 (95% CI: 0.373–0.618)<br>*Algorithm D*<br>*Agreement*: 52.2 (95% CI: 42.0–62.2)<br>kappa score: 0.378 (95% CI: 0.214–0.497)<br>*Algorithm E*<br>*Agreement*: 52.2 (95% CI: 42.0–62.2)<br>kappa score: 0.384 (95% CI: 0.278–0.497)<br>*Algorithm F*<br>*Agreement*: 64.4 (95% CI: 54.1–73.6)<br>kappa score: 0.526 (95% CI: 0.387–0.627)<br>*Algorithm G*<br>*Agreement*: 78.9 (95% CI: 69.4–86.0)<br>kappa score: 0.722 (95% CI: 0.613–0.828)		

(Diamond et al., 2015)	Prospective, double-blinded, multi-center study	Embryo selection	54 patients	758 day 3 embryos	Embryo assessment using morphology followed by Eeva test results	Embryo assessment using morphology alone	Blastocyst formation rate (odds ratio)	3.51 vs 2.69	95% CI: 2.62–4.69 vs 2.06–3.50)	
(Manna et al., 2013)	Retrospective study	Embryo selection	104 patients	269 images of oocytes and 269 images of the corresponding embryos recorded with an inverted microscope	Levenberg-Marquardt neural networks	Other machine learning methods	Prediction of the quality of embryos and oocytes	The artificial intelligence system proposed in this work is based on a set of Levenberg-Marquardt neural networks trained using textural descriptors (the local binary patterns). The proposed system was tested on two data sets of 269 oocytes and 269 corresponding embryos from 104 women and compared with other machine learning methods already proposed in the past for similar classification problems		

Trigger injection timing

REFERENCES	STUDY DESIGN	TECHNIQUE	POPULATION	SAMPLE SIZE	INTERVENTION	COMPARISON	OUTCOME VARIABLES	RESULTS	MEAN DIFFERENCE	P
(Hariton et al., 2021)<br><br>Optimize trigger injection timing to maximize: the yield of fertilized oocytes (2PNs) and the total usable blastocysts	Retrospective study	Intracytoplasmic sperm injection	7,866 patients from 2008 to 2019 at the University of California San Francisco Center for Reproductive Health		Machine learning algorithm	Physician’s decision	Optimize the yield of fertilized oocytes (2PNs)	Average outcome improvement: 3.015	95% CI: 2.626, 3.371	-
							Optimize the total usable blastocysts	Average outcome improvement: 1.515	95% CI: 1.134, 1.871	

FSH starting dose

REFERENCES	STUDY DESIGN	TECHNIQUE	POPULATION	SAMPLE SIZE	INTERVENTION	COMPARISON	OUTCOME VARIABLES	RESULTS	MEAN DIFFERENCE	P
(Correa et al., 2022)	Retrospective study	Starting dose	NA	2,713 patients	FSH dose calculator	Clinician's prescription	Predict the initial dose of FSH to achieve a number of MII oocytes as close as possible to 12	The model reached a mean performance score of 0.87 (95% CI: 0.86 to 0.88) in the development phase, significantly better than for doses prescribed by clinicians for the same patients (0.83, 95% CI: 0.82 to 0.84)		2.44 e-10
							Test whether the recommendations made by the model are better than the prescriptions made by the clinicians	Mean performance score of the model recommendations was 0.89 (95% CI: 0.88 to 0.90) in the validation phase, also significantly better than clinicians (0.84, 95% CI: 0.82 to 0.86)		3.81 e-05
(Olivennes et al., 2015)	RCT<br><br>Prospective, randomized, controlled, multiregional, open-label, phase IV study (NCT00829244)	Ovarian stimulation	Normo-ovulatory women (aged 18–34 years from 23 centres (nine European countries and Chile)	200 women (96: CONSORT; 104: starting dosing)	CONSORT calculator	Follitropin alfa starting dose (150IU)	Number of oocytes retrieved per patient	10 (5.6) vs 11.8 (5.3)<br>Estimated difference (SE): −1.7 (0.8); 95% CI: −3.3, −0.1		0.037
							Secondary: mean total and daily doses of rFSH	Total dose of rFSH (IU): 1,288.5 (301.0) vs 1,810.0 (546.9) Estimated difference: −511.4 (64.5); 95% CI: −638.8, −384.0; P<0.001<br><br>Daily dose of rFSH (IU): 121.5 (22.6) vs 167.4 (30.8) Estimated difference: −45.6 (4.1); 95% CI: −53.8, 37.5; P< 0.001		
							Duration of ovarian stimulation	10.6 (1.7) vs 10.7 (1.6)<br>Estimated difference: 0.0 (0.2); 95% CI: −0.4, 0.5; NS		

							Number of embryos transferred	1.6 (0.7) vs 1.7 (0.8)<br>Estimated difference (SE): 0.0 (0.1); 95% CI: −0.2 to 0.1		
							Number of embryos transferred and cryopreserved	Mean [SD]: 2.2 [2.7] vs 2.6 [3.3] embryos; Estimated difference (SE): −0.2 [0.4]; 95% CI: −1.0 to 0.5		
							Clinical pregnancy rates	36.0% vs 35.5%<br>Estimated difference: 0.6%; 95% CI: −13.5, 14.6		
							Multiple pregnancy rates	6 vs 9		
							Implantation rate	31.1% vs 31.2%<br>Estimated difference: 0.6% (6.6); 95% CI: −12.3, 13.6; NS		
							Cycle cancellation rate	Resulting from insufficient ovarian response: 9.4% [9/96] vs 4.8% [5/104]<br>Resulting from an excessive ovarian response: 5.8% [6/104] vs 1.0% [1/96]		

Other

REFERENCES	STUDY DESIGN	TECHNIQUE	POPULATION	SAMPLE SIZE	INTERVENTION	COMPARISON	OUTCOME VARIABLES	RESULTS	MEAN DIFFERENCE	P
(Yuan et al., 2023)	Retrospective study	Prediction of risk of missed abortion	NA	1,017 patients	XGBoost algorithm	Logical regression model	Predict risk of missed abortion	Training set AUC: 0.877±0.014 vs 0.713±0.013 F1 score: 0.730±0.019 vs 0.568±0.026 Test set AUC: 0.759±0.023 vs 0.695±0.030 F1 score: 0.566±0.042 vs 0.550±049		
(Barnett-Itzhaki et al., 2020)	Retrospective study	Prediction of IVF outcomes	NA	136 patients	Machine learning algorithms (support vector machine, SVM; artificial neural network, NN)	Classic statistic (logistic regression)	Number of retrieved oocytes	SVM/NN vs Logistic regression F1 score: 0.45/0.69 vs 0.35 Accuracy: 0.46/0.69 vs 0.34		
							Number of mature oocytes	F1 score: 0.74/0.87 vs 0.74 Accuracy: 0.77/0.88 vs 0.74		
							Number of fertilized oocytes	F1 score: 0.60/0.77 vs 0.56 Accuracy: 0.59/0.77 vs 0.55		
							Number of top-quality embryos	F1 score: 0.64/0.85 vs 0.60 Accuracy: 0.63/0.86 vs 0.61		
							Positive beta-hCG	F1 score: 0.60/0.84 vs 0.43 Accuracy: 0.59/0.85 vs 0.53		
							Clinical pregnancy	F1 score: 0.70/0.89 vs 0.46 Accuracy: 0.63/0.90 vs 0.58		
							Live birth	F1 score: 0.74/0.86 vs 0.36		
(Blank et al., 2019)	Retrospective study	Prediction of implantation after blastocyst transfer	NA	1,052 patients	Random forest model (RFM)	Multivariate logistic regression model (MvLRM)	Predict pregnancy	AUC: 0.74±0.03 vs 0.66±0.05	AUC increase from 0.66±0.05 to 0.74±0.05	<0.001

References

Ahlström, A., Lundin, K., Lind, A.-K., Gunnarsson, K., Westlander, G., Park, H., Thurin-Kjellberg, A., Thorsteinsdottir, S. A., Einarsson, S., Åström, M., Löfdahl, K., Menezes, J., Callender, S., Nyberg, C., Winerdal, J., Stenfelt, C., Jonassen, B.-R., Oldereid, N., Nolte, L., … Hardarson, T. (2022). A double-blind randomized controlled trial investigating a time-lapse algorithm for selecting Day 5 blastocysts for transfer. *Human Reproduction (Oxford, England)*, *37*(4), 708–717. https://doi.org/10.1093/humrep/deac020

Barnett-Itzhaki, Z., Elbaz, M., Butterman, R., Amar, D., Amitay, M., Racowsky, C., Orvieto, R., Hauser, R., Baccarelli, A. A., & Machtinger, R. (2020). Machine learning vs. Classic statistics for the prediction of IVF outcomes. *Journal of Assisted Reproduction and Genetics*, *37*(10), 2405–2412. https://doi.org/10.1007/s10815-020-01908-1

Berntsen, J., Rimestad, J., Lassen, J. T., Tran, D., & Kragh, M. F. (2022). Robust and generalizable embryo selection based on artificial intelligence and time-lapse image sequences. *PloS One*, *17*(2), e0262661. https://doi.org/10.1371/journal.pone.0262661

Blank, C., Wildeboer, R. R., DeCroo, I., Tilleman, K., Weyers, B., de Sutter, P., Mischi, M., & Schoot, B. C. (2019). Prediction of implantation after blastocyst transfer in in vitro fertilization: A machine-learning perspective. *Fertility and Sterility*, *111*(2), 318–326. https://doi.org/10.1016/j.fertnstert.2018.10.030

Bodri, D., Milewski, R., Yao Serna, J., Sugimoto, T., Kato, R., Matsumoto, T., & Kawachiya, S. (2018). Predicting live birth by combining cleavage and blastocyst-stage time-lapse variables using a hierarchical and a data mining-based statistical model. *Reproductive Biology*, *18*(4), 355–360. https://doi.org/10.1016/j.repbio.2018.10.006

Chavez-Badiola, A., Flores-Saiffe-Farías, A., Mendizabal-Ruiz, G., Drakeley, A. J., & Cohen, J. (2020). Embryo Ranking Intelligent Classification Algorithm (ERICA): Artificial intelligence clinical assistant predicting embryo ploidy and implantation. *Reproductive Biomedicine Online*, *41*(4), 585–593. https://doi.org/10.1016/j.rbmo.2020.07.003

Correa, N., Cerquides, J., Arcos, J. L., & Vassena, R. (2022). Supporting first FSH dosage for ovarian stimulation with machine learning. *Reproductive Biomedicine Online*, *45*(5), 1039–1045. https://doi.org/10.1016/j.rbmo.2022.06.010

Diamond, M. P., Suraj, V., Behnke, E. J., Yang, X., Angle, M. J., Lambe-Steinmiller, J. C., Watterson, R., Athayde Wirka, K., Chen, A. A., & Shen, S. (2015). Using the Eeva Test™ adjunctively to traditional day 3 morphology is informative for consistent embryo assessment within a panel of embryologists with diverse experience. *Journal of Assisted Reproduction and Genetics*, *32*(1), 61–68. https://doi.org/10.1007/s10815-014-0366-1

Enatsu, N., Miyatsuka, I., An, L. M., Inubushi, M., Enatsu, K., Otsuki, J., Iwasaki, T., Kokeguchi, S., & Shiotani, M. (2022). A novel system based on artificial intelligence for predicting blastocyst viability and visualizing the explanation. *Reproductive Medicine and Biology*, *21*(1), e12443. https://doi.org/10.1002/rmb2.12443

Feyeux, M., Reignier, A., Mocaer, M., Lammers, J., Meistermann, D., Barrière, P., Paul-Gilloteaux, P., David, L., & Fréour, T. (2020). Development of automated annotation software for human embryo morphokinetics. *Human Reproduction (Oxford, England)*, *35*(3), 557–564. https://doi.org/10.1093/humrep/deaa001

Fishel, S., Campbell, A., Foad, F., Davies, L., Best, L., Davis, N., Smith, R., Duffy, S., Wheat, S., Montgomery, S., Wachter, A., & Beccles, A. (2020). Evolution of embryo selection for IVF from subjective morphology assessment to objective time-lapse algorithms improves chance of live birth. *Reproductive Biomedicine Online*, *40*(1), 61–70. https://doi.org/10.1016/j.rbmo.2019.10.005

Fitz, V. W., Kanakasabapathy, M. K., Thirumalaraju, P., Kandula, H., Ramirez, L. B., Boehnlein, L., Swain, J. E., Curchoe, C. L., James, K., Dimitriadis, I., Souter, I., Bormann, C. L., & Shafiee, H. (2021). Should there be an “AI” in TEAM? Embryologists selection of high implantation potential embryos improves with the aid of an artificial intelligence algorithm. *Journal of Assisted Reproduction and Genetics*, *38*(10), 2663–2670. https://doi.org/10.1007/s10815-021-02318-7

Friedenthal, J., Hernandez-Nieto, C., Roth, R. M., Slifkin, R., Gounko, D., Lee, J. A., Nazem, T., Briton-Jones, C., & Copperman, A. (2021). Clinical implementation of algorithm-based embryo selection is associated with improved pregnancy outcomes in single vitrified warmed euploid embryo transfers. *Journal of Assisted Reproduction and Genetics*, *38*(7), 1647–1653. https://doi.org/10.1007/s10815-021-02203-3

Hariton, E., Chi, E. A., Chi, G., Morris, J. R., Braatz, J., Rajpurkar, P., & Rosen, M. (2021). A machine learning algorithm can optimize the day of trigger to improve in vitro fertilization outcomes. *Fertility and Sterility*, *116*(5), 1227–1235. https://doi.org/10.1016/j.fertnstert.2021.06.018

Herbert, S.-L., Staib, C., Wallner, T., Löb, S., Curtaz, C., Schwab, M., Wöckel, A., & Häusler, S. (2023). Morphokinetic analysis of early human embryonic development and its relationship to endometriosis resection: A retrospective time-lapse study using the KIDScore™ D3 and D5 implantation data algorithm. *Archives of Gynecology and Obstetrics*, *308*(2), 587–597. https://doi.org/10.1007/s00404-023-07008-6

Khosravi, P., Kazemi, E., Zhan, Q., Malmsten, J. E., Toschi, M., Zisimopoulos, P., Sigaras, A., Lavery, S., Cooper, L. A. D., Hickman, C., Meseguer, M., Rosenwaks, Z., Elemento, O., Zaninovic, N., & Hajirasouliha, I. (2019). Deep learning enables robust assessment and selection of human blastocysts after in vitro fertilization. *NPJ Digital Medicine*, *2*, 21. https://doi.org/10.1038/s41746-019-0096-y

Kovacs, P., Matyas, S., Forgacs, V., Sajgo, A., Molnar, L., & Pribenszky, C. (2019). Non-invasive embryo evaluation and selection using time-lapse monitoring: Results of a randomized controlled study. *European Journal of Obstetrics, Gynecology, and Reproductive Biology*, *233*, 58–63. https://doi.org/10.1016/j.ejogrb.2018.12.011

Kragh, M. F., Rimestad, J., Berntsen, J., & Karstoft, H. (2019). Automatic grading of human blastocysts from time-lapse imaging. *Computers in Biology and Medicine*, *115*, 103494. https://doi.org/10.1016/j.compbiomed.2019.103494

Manna, C., Nanni, L., Lumini, A., & Pappalardo, S. (2013). Artificial intelligence techniques for embryo and oocyte classification. *Reproductive Biomedicine Online*, *26*(1), 42–49. https://doi.org/10.1016/j.rbmo.2012.09.015

Olivennes, F., Trew, G., Borini, A., Broekmans, F., Arriagada, P., Warne, D. W., & Howles, C. M. (2015). Randomized, controlled, open-label, non-inferiority study of the CONSORT algorithm for individualized dosing of follitropin alfa. *Reproductive Biomedicine Online*, *30*(3), 248–257. https://doi.org/10.1016/j.rbmo.2014.11.013

Rocafort, E., Enciso, M., Leza, A., Sarasa, J., & Aizpurua, J. (2018). Euploid embryos selected by an automated time-lapse system have superior SET outcomes than selected solely by conventional morphology assessment. *Journal of Assisted Reproduction and Genetics*, *35*(9), 1573–1583. https://doi.org/10.1007/s10815-018-1265-7

Sawada, Y., Sato, T., Nagaya, M., Saito, C., Yoshihara, H., Banno, C., Matsumoto, Y., Matsuda, Y., Yoshikai, K., Sawada, T., Ukita, N., & Sugiura-Ogasawara, M. (2021). Evaluation of artificial intelligence using time-lapse images of IVF embryos to predict live birth. *Reproductive Biomedicine Online*, *43*(5), 843–852. https://doi.org/10.1016/j.rbmo.2021.05.002

Storr, A., Venetis, C., Cooke, S., Kilani, S., & Ledger, W. (2018). Time-lapse algorithms and morphological selection of day-5 embryos for transfer: A preclinical validation study. *Fertility and Sterility*, *109*(2), 276-283.e3. https://doi.org/10.1016/j.fertnstert.2017.10.036

Targosz, A., Przystałka, P., Wiaderkiewicz, R., & Mrugacz, G. (2021). Semantic segmentation of human oocyte images using deep neural networks. *Biomedical Engineering Online*, *20*(1), 40. https://doi.org/10.1186/s12938-021-00864-w

Ueno, S., Berntsen, J., Ito, M., Uchiyama, K., Okimura, T., Yabuuchi, A., & Kato, K. (2021). Pregnancy prediction performance of an annotation-free embryo scoring system on the basis of deep learning after single vitrified-warmed blastocyst transfer: A single-center large cohort retrospective study. *Fertility and Sterility*, *116*(4), 1172–1180. https://doi.org/10.1016/j.fertnstert.2021.06.001

VerMilyea, M., Hall, J. M. M., Diakiw, S. M., Johnston, A., Nguyen, T., Perugini, D., Miller, A., Picou, A., Murphy, A. P., & Perugini, M. (2020). Development of an artificial intelligence-based assessment model for prediction of embryo viability using static images captured by optical light microscopy during IVF. *Human Reproduction (Oxford, England)*, *35*(4), 770–784. https://doi.org/10.1093/humrep/deaa013

Yuan, G., Lv, B., Du, X., Zhang, H., Zhao, M., Liu, Y., & Hao, C. (2023). Prediction model for missed abortion of patients treated with IVF-ET based on XGBoost: A retrospective study. *PeerJ, 11*, e14762. https://doi.org/10.7717/peerj.14762
