# Supplementary material for: Artificial Intelligence, Clinical Decision Support Algorithms, Mathematical Models, Calculators Applications in Infertility: Systematic Review and Hands-On Digital Applications
Source: Mayo Clin Proc Digit Health. 2024 Aug 26;2(4):518–32. doi: 10.1016/j.mcpdig.2024.08.007 (PMC11975849; doi:10.1016/j.mcpdig.2024.08.007)
Supplement: Supplementary Table 2 [file mmc2.pdf]

Embryo quality

REFERENCES	STUDY DESIGN	TECHNIQUE	POPULATION	SAMPLE SIZE	INTERVENTION	COMPARISON	OUTCOME VARIABLES	RESULTS	MEAN DIFFERENCE	P
(Diakiw *et al.*, 2022)	Retrospective study	Embryo quality	4,709 patients who received a single embryo transfer with a day 5 blastocyst-stage embryo	9,359 two-dimensional embryo images (standard or time-lapse-based optical light microscopy systems)	AI-based model		Predict clinical pregnancy outcome	ROC-AUC: 0.68		
(Zhao *et al.*, 2021)	Retrospective study	Embryo quality	14 patients	1,218 day-one embryo time-lapse images	Convolutional neural network (CNN) algorithm		Test the precision of segmentation	The precisions of segmentation were that cytoplasm over 97%, PN over 84% and ZP around 80%		
(Coticchio *et al.*, 2021)	Retrospective study	Embryo quality	NA	230 preimplantation embryos	Artificial neural network		Predict blastocyst development	Accuracy: 75.4%<br>Sensitivity: 76.5%<br>Specificity: 74.3%<br>Precision: 74.3%<br>F1 score: 75.4%		
(Bamford *et al.*, 2023)	Retrospective study	Embryo quality	1,725 patients	8,147 blastocysts	Machine learning methods		Predict blastocyst ploidy status	The best-performing model was logistic regression built using the larger dataset with 22 predictors (F1 score: 0.59 for predicting euploidy; F1 score: 0.77 for predicting aneuploidy; AUC: 0.71; 95% CI: 0.67–0.73). The best-performing models using the algorithms from random forest, extreme gradient boosting, and deep learning achieved an AUC: 0.68, 0.63, and 0.63, respectively		
(Zieliński *et al.*, 2023)	Retrospective study	Embryo selection	NA	6,043 patients	Machine learning model based on the gradient boosting machine (GBM) technique implemented by the LightGBM framework		Personalized prediction of the secondary oocytes number after ovarian stimulation based on clinical data			

(Barnes *et al.*, 2023)	Retrospective study	Embryo selection	1,385 patients	10,378 embryos	AI-base model (STORK-A)		Predict blastocyst aneuploid	Accuracy: 69,3% (95% CI: 66,9-71,5)<br>AUC: 0,761<br>Positive predictive value: 76,1%<br>Negative predictive value: 62,1%		
(Fukunaga *et al.*, 2020)	Retrospective study	Embryo selection	NA	Time-lapse images from 900 embryos judged by embryologists as 0PN, 1PN, and 2PN from the pronuclear stage	Deep learning techniques	Embryologists	Automated pronuclei determination	The sensitivity rates of 0PN, 1PN, and 2PN were 99%, 82%, and 99%, respectively.		
(Ferrand *et al.*, 2023)	Retrospective study	Oocytes selection	NA	11,286 cycles	Light Gradient Boosting Machine algorithm		Predict the number of oocytes retrieved from controlled ovarian hyperstimulation			
(Huang *et al.*, 2022)	Retrospective study	Embryo selection	5,913 patients	33,738 time lapse images	Deep learning model		Predict outcome of live birth	AUC: 0.968 (in 5-fold stratified cross-validation)		
(Huang *et al.*, 2021a)	Retrospective study	Embryo selection	469 patients	469 preimplantation genetic testing cycles and time lapse images from 1803 blastocysts	Euploid prediction algorithm (EPA)		Predict embryo ploidy status	AUC: 0.80		
(Lee *et al.*, 2021)	Retrospective study	Embryo selection	108 patients	690 sets of time-lapse images with known outcome of preimplantation genetic testing for aneuploidy (training set: 80%; testing set: 20%)	End-to-end deep learning model		Identify ploidy status	AUC: 0.74		
(Blais *et al.*, 2021)	Retrospective study	Embryo selection	1,075 patients	1,879 embryos with known implantation data (KID). (KID-positive: 425; KID-negative: 1,454)	Predictive algorithm developed used the EmbryoScope Stats software		Discriminate between KID-positive and KID-negative day-2, -3, and -5 embryos	AUC<br>*Day-2 embryos*: 0.657<br>*Day-3 embryos*: 0.673<br>*Day-5 embryos*: 0.803		
(Liao *et al.*, 2021)	Retrospective study	Embryo selection	NA	750–800 time lapse images (training set: 80%; validation set: 20%)	Ensemble prediction models (STEM and STEM+)		Blastocysts formation	STEM model<br>*Accuracy*: 78.2%<br>*AUC*: 0.82		
							Usable blastocysts	STEM+ model<br>*Accuracy*: 71.9%<br>*AUC*: 0.79		
(Thirumalaraju *et al.*, 2021)	Retrospective study	Embryo selection	543 patients	2,440-time lapse images of day 5	Multiple convolutional neural networks		Embryo classification	Accuracy/SD<br>Xception: 90.48%±0.273%		

				embryo (113 h post insemination) (training set: 1,188; validation set: 510; independent non-overlapping test set: 742)	(CNNs) (Inception v3, ResNET-50, Inception-ResNET-v2, NASNetLarge, ResNeXt-101, ResNeXt-50, and Xception)		(blastocyst/non-blastocyst)	ResNET-50: 89.08%±0.812%<br>Inception-v3: 89.71%±0.72%<br>NASNetLarge: 78.44%±0.233%<br>Multilayer CNN: 82.2%±0.546%<br>ResNeXt-101: 90.75%±0.273%<br>ResNeXt-50: 89.94%±0.574%<br>Inception-ResNET-v2: 90.21%±0.518%		
(Geller *et al.*, 2021)	Retrospective study	Embryo selection		361 static images	Convolutional neural network (CNN)		Predict pregnancy based on static images captured by optical light microscopy	AUC: 0.657		
(Xi *et al.*, 2021)	Retrospective study	Embryo selection	9,211 patients	10,076 embryos	Machine learning model (XGBoost)		Predict single-embryo transfer pregnancy	AUC: 0.7945		
							Predict double-embryo transfer pregnancy	AUC: 0.8385		
							Predict twin risk	AUC: 0.7229		
(Giscard d'Estaing *et al.*, 2021)	Retrospective study	Embryo selection	311 couples (training data group: 110; global setting data group: 201)	2,077 embryos (training data group: 891; global setting data group: 1,186)	Machine learning system (DynScore)		Predict blastocyst formation based on morphokinetic parameters	Training data group AUC: 0.634<br>Global setting data AUC: 0.638		<0.001<br><0.001
(Bori *et al.*, 2020)	Retrospective study	Embryo selection	637 patients from the oocyte donation program who underwent single-blastocyst transfer during two consecutive years	451 embryos	Artificial neural network (ANN) algorithms		Predict implantation	AUC<br>*ANN1 (conventional morphokinetics)*: 0.64<br>*ANN2 (novel morphodynamics)*: 0.73<br>*ANN3 (conventional morphokinetics + novel morphodynamics)*: 0.77<br>*ANN4 (discriminatory variables from statistical test):* 0.68		

(Tran *et al.*, 2019)	Retrospective study	Embryo selection	1,648 patients	10,638 embryos	Time-lapse videos. Deep learning model (IVY)		Predict the probability of pregnancy with fetal heart (FH)	AUC: 0.93	95% CI: 0.92-0.94	
(Kanakasabapathy *et al.*, 2019)	Retrospective study	Embryo selection	NA	272 embryo images recorded on the stand-alone and 319 embryo images recorded on smartphone-based optical systems	Automated imaging platforms		Embryo classification (blastocyst/non-blastocyst) STAND ALONE SYSTEM	AUC: 0.98	CI: 0.95 to 0.99	
								Accuracy: 96.69%	CI: 93.81 to 98.48%	
								Sensitivity: 95.31%	CI: 91.29 to 97.83%	
								Specificity: 100%	CI: 95.49 to 100%	
								Positive predictive value: 100%		
								Negative predictive value: 89.89%	CI: 82.45 to 94.39%	
							Embryo classification (blastocyst/non-blastocyst) SMARTPHONE OPTICAL SYSTEM	AUC: 0.94	CI: 0.90 to 0.96	
								Accuracy: 92.16%	CI: 88.65 to 94.86%	
								Sensitivity: 98.54%	CI: 95.78 to 99.70%	
								Specificity: 80.70%	CI: 72.25 to 87.49%	
								Positive predictive value: 90.18%	CI: 86.31% to 93.04%	
								Negative prediction value: 96.84%	CI: 90.86% to 98.95%	
(Reignier *et al.*, 2019)	Retrospective study	Embryo selection	NA	5 day embryos	KIDScore™ Day 5 version 1 and version 2		Implantation rate	Positive correlation between KIDScore™ Day 5 v1 and implantation rate (r: 0.96, p=0.001)<br><br>Positive correlation between KIDScore™ Day 5 v2 and implantation rate (r: 0.90, p= 0.01)		
							Prediction of implantation (ROC curve analysis)	KIDScore™ Day 5 v1 AUC: 0.59	95% CI: 0.52–0.66	0.02
								KIDScore™ Day 5 v2 AUC: 0.60	95% CI: 0.51–0.67;	0.005
							blastocyst expansion rate ROC curve analysis	AUC: 0.60	95% CI: 0.52–0.67	0.018
							Blastocyst morphology score	AUC: 0.61	95% CI: 0.54–0.69	0.004

							ROC curve analysis			
							Concordance with the embryologist (based on morphology)	KIDScore™ day 5 v1: 78%<br>KIDScore™ day 5 v2: 61.4%		
(Liu *et al.*, 2019) ok	Retrospective study		Fertility North between February 2013 and December 2014	Retrospectively generated datasets:<br>(a) KID dataset (n=270)<br>(b) a subset of SET (n=144, end-point: implantation)<br>(c) SET (n=144, end-point: live birth)	Four published day 3 embryo time-lapse algorithms based on different types of datasets (known implantation data [KID] and single embryo transfer [SET]) and the confounding effect of female age and conventional embryo morphology			All four algorithms showed progressively reduced predictive power (expressed as area under the receiver operating characteristics curve and 95% CI) after application to the three datasets (a-c)		
(Dirvanauskas *et al.*, 2019)	Retrospective study	Embryo selection	NA	7,002 time lapse images	Embryo image Classification Algorithm (EMCA) based on Convolutional Neural Network (CNN)		Embryo development stage	Overall accuracy: 97.62%		
(Alegre *et al.*, 2019)					To develop a noninvasive embryo selection algorithm consisting of time-lapse morphokinetics and the oxidative status of the spent embryo culture medium determined using the Thermochemiluminescence (TCL) Analyzer.					
(Adolfsson *et al.*, 2018)	Retrospective study	Embryo selection	NA	768 embryos with known outcome between 2013 - 2015	To validate a morphokinetic implantation model developed for EmbryoScope on embryos with known outcome, compared to standard morphology in a			Morphokinetic annotation of 768 embryos with known outcome between 2013 - 2015; corresponding to 116 D3 fresh embryos, 80 D6 frozen blastocysts,		

					retrospective single center study.			and 572 D5 blastocysts, fresh or frozen. The embryos were ranked by the KIDScore into five classes, KID1-5, and grouped into four classes based on standard morphology. Pregnancy rates, clinical pregnancy rates and live birth rates were compared. Combinations of morphology and morphokinetics were evaluated for implantation rates and live births.		
(Strouthopoulos and Anifandis, 2018)<br>An automated blastomere identification method for the evaluation of day 2 embryos during IVF/ICSI treatments	Retrospective study	Embryo selection	NA	2D grayscale image of day-2 embryo	Algorithm for automatic zona pellucida thickness identification		Identify the most suitable cleaving embryos			
(Milewski *et al.*, 2017)	Retrospective study	Embryo selection	NA	Time-lapse images of 610 embryos	Artificial neural networks (ANN) and principal component analysis (PCA)		Predict embryo implantation based on morphokinetic informations	AUC: 0.71	95% CI: 0.59-0.84	
(Petersen *et al.*, 2016)	Retrospective study	Embryo selection	NA	Data extracted from a database of known implantation data (KID) originating from 3,275 embryos transferred on Day 3	Morphokinetic algorithm (KIDScore)		Predict implantation rate based on	AUC: 0.650		
							Predict blastocyst development	AUC: 0.745		
							Predict blastocyst quality	AUC: 0.679		
(VerMilyea *et al.*, 2014)	Multicenter study	Embryo selection	NA	205 patients whose embryos were imaged by the EevaTM System.	Eeva System		Implantation rate	Embryos with high, medium and low scores:<br>*High vs Low*: 37% vs 15%		<0.0001<br><br>0.0004

								Medium vs Low: 35% vs 15%		
							Clinical pregnancy rate	Patients with at least one High embryo, transferred, however, had statistically significantly higher clinical pregnancy rates than those with no High embryos transferred 51% vs 39%		0.04
(Morales *et al.*, 2008)	Retrospective study	Embryo selection	NA	189 embryos	Bayesian classification models		Implantation rate	Implantation predictive value: *naive Bayes*: 43.75 *selective naive Bayes:* 50 *semi naive Bayes:* 25 *tree augmented naive Bayes (TAN):* 14.29 *k-dependence Bayesian classifier (kDB):* 11.11		

Sperm analysis

REFERENCES	STUDY DESIGN	TECHNIQUE	POPULATION	SAMPLE SIZE	INTERVENTION	COMPARISON	OUTCOME VARIABLES	RESULTS	MEAN DIFFERENCE	P
(Valiuškaitė *et al.*, 2020)	Retrospective study	Sperm analysis Sperm head motility	85 patients	30 GB of videos	Region Based Convolutional Neural Networks (R-CNN) architecture		Accuracy	91.77%	95% CI: 91.11–92.43%	
							Mean absolute error	2.92	95% CI: 2.46-3.37	
							Pearson correlation coefficient between sperm vitality established using laboratory analysis methods and predicted	0.969	95% CI: 0.968–0.97	<0.001
(Yi *et al.*, 1998)	Retrospective study	Sperm analysis	Sperm morphological classification using artificial neural networks -pattern recognition and image processing. multi-layer perceptron trained by the error back-propagation algorithm							
(Ottl *et al.*, 2022)	Retrospective study	Sperm analysis		85 videos of live spermatozoa (Visem dataset)	Machine learning methods		Predict sperm motility	Mean absolute error:<br>SVR: 8.60<br>MLP: 8.83		
(Saiffe Farías *et al.*, 2022)		Sperm analysis								
(Javadi and Mirroshandel, 2019)	Retrospective study	Sperm analysis	235 patients	1,540 sperm images	Deep learning algorithm		Detection of sperm morphology malformations	F0.5 scores for acrosome abnormality (84.74%), head abnormality (83.86%), vacuole abnormality (94.65%)		
(Abbasi *et al.*, 2021)	Retrospective study	Sperm analysis	NA	1,540 sperm images (MHSMA dataset) (test set: 308; validation set: 1,232)	Deep transfer learning (DTL)	Deep multi-task transfer learning (DMTL),	Detection of sperm morphology malformations	Head:<br>Accuracy: 84% vs 82%;<br>F0.5 score: 87.92 vs 84.89<br>ROC-AUC: 81.56 vs 78.40<br><br>Vacuole:<br>Accuracy: 94% vs 92.33%		

								F0.5 score: 95.75 vs 95.11<br>ROC-AUC: 96.62 vs 95.65<br><br>Acrosome:<br>Accuracy: 79% vs 80.66%<br>F0.5 score: 82.57 vs 84.26<br>ROC-AUC: 79.65 vs 78.19		
(Jiang *et al.*, 2022)	Retrospective study	Sperm analysis	NA	1,471 images of immotile sperm	AI-based method		Predict viability of immotile sperm	Accuracy: 94.9<br>Recall: 97.0%<br>Specificity: 93.3%		
(McCallum *et al.*, 2019)	Retrospective study	Sperm analysis	NA	1,064 bright-field sperm cell images	Deep convolutional neural network		Predict sperm DNA quality	Our results demonstrate moderate correlation (bivariate correlation ~0.43) between a sperm cell image and DNA quality and the ability to identify higher DNA integrity cells relative to the median.		
(Peng *et al.*, 2023)	Retrospective study	Sperm quality assessment	NA	1,258 couples	Machine learning-based clustering		Identify the combined effect of the DNA fragmentation index and conventional semen parameters on IVF outcomes			
(Mendizabal-Ruiz *et al.*, 2022)	Retrospective study	Sperm selection	NA	383 individual spermatozoa from 78 ICSI cycles	Artificial vision-based software (SiD V1.0; IVF 2.0)		Successful fertilization and blastocyst formation	Statistically significant differences (P=0.004) were observed between the mean SiD scores of spermatozoa with a positive fertilization outcome (0.51±0.10) and those with a negative outcome (0.43±0.12). Similarly,		

								statistically significant differences (P=0.013) were found between the SiD scores of those spermatozoa that were associated with blastocyst generation (0.52±0.10) and those that did not (0.46±0.11).		

Predict day of triggering

REFERENCES	STUDY DESIGN	TECHNIQUE	POPULATION	SAMPLE SIZE	INTERVENTION	COMPARISON	OUTCOME VARIABLES	RESULTS	MEAN DIFFERENCE	P
(Letterie *et al.*, 2022)	Retrospective study	Predict day of triggering	NA	1,591 autologous cycles	First-iteration algorithm		Identify the single best day for monitoring and predict trigger day options and total number of oocytes retrieved	The mean error to predict the single best day for monitoring was 1.355 days. Accuracy for prediction of total number of oocytes with baseline testing alone or in combination with data on the day of observation was 0.76 and 0.80, respectively. The sensitivities for estimating the total number and number of mature oocytes based solely on pre-IVF profiles in group I (0-10) were 0.76 and 0.78, and in group II (>10) 0.76 and 0.81, respectively.		

IVF outcome

REFERENCES	STUDY DESIGN	TECHNIQUE	POPULATION	SAMPLE SIZE	INTERVENTION	COMPARISON	OUTCOME VARIABLES	RESULTS	MEAN DIFFERENCE	P
(Qiu *et al.*, 2019)	Retrospective study	Predict IVF outcome	7,188 women at their first IVF		Machine-learning based model (XGBoost)		Predict live birth chance	The XGBoost model achieved an area under the ROC curve of 0.73 on the validation dataset and showed the best calibration compared with other machine learning algorithms. Nested cross-validation resulted in an average accuracy score of 0.70±0.003 for the XGBoost model.		
(Bardet *et al.*, 2022)	Retrospective cohort study	Predict IVF outcome	NA	3,045 couples	Cox regression	Tree ensemble-based model using XGBoost implementation	Predict the cumulative live birth rate	C-index: 58% vs 60%		
(Fu *et al.*, 2022)	Retrospective study	Predict IVF outcome	NA	37,062 cycles	Gradient boosting decision tree (GBDT)		Predict the clinical pregnancy rate	AUC: 0.704<br>Consistency: 98.1%		
(Qu *et al.*, 2022)		Predict IVF outcome	NA	26,689 patients (training set: 18601 patients; validation set: 8088 patients)	Nomogram		Predict cumulative live birth	Training group: AUC: 0.676<br>Validation group: AUC: 0.672	Training group: 95% CI: 0.668 to 0.684<br>Validation group: 95% CI: 0.660 to 0.684	
(Wen *et al.*, 2022)	Retrospective study	Predict IVF outcome	NA	1,329 cycles	Machine learning models (logistic regression; random forest; SVM; LightGBM; MLP; XGBoost)		Predict the pregnancy outcome and multiple pregnancy risk	AUC<br>Logistic regression: 0.783<br>Random forest: 0.769<br>SVM: 0.781<br>LightGBM 0.752<br>MLP: 0.762<br>XGBoost: 0.787		
(Gong *et al.*, 2023)	Retrospective study	Predict IVF outcome	NA	657 poor ovarian response patients (training set: 438; validation set: 219)	Nomogram based on POSEIDON criteria		Predict live birth rate	AUC<br>Training set: 0.820<br>Validation set: 0.879		
(Jones *et al.*, 2011)	Retrospective study	Predict IVF outcomes	NA	14,167 IVF cycles	Embryology Authority (HFEA) metrics [Software-as-a-Service (SaaS)]		Predict live birth rate and probability of multiple birth			

(Canosa *et al.*, 2024)	Retrospective study	Prediction of blastocyst development	575 embryos obtained from 80 women		Machine learning combined with timelapse technology		Predictive power (embryos to develop at blastocyst stage on day 5 based on Integrated Morphology Cleavage Score (IMCS)	AUC: 0.84<br>Accuracy: 81%		
(Liu *et al.*, 2020)	Retrospective study	Prediction of early pregnancy loss based on fetal heart rate	NA	31,030 cases at 6–10 weeks' gestation		Prediction model based on machine learning algorithms [Logistic Regression (LR), Support Vector Machine (SVM), Decision Tree (DT), Back Propagation Neural Network (BNN), XGBoost and Random Forest (RF)]	Predict early pregnancy loss	Accuracy: 97%<br>AUC: 0.970		
(Uyar *et al.*, 2015)	Retrospective cohort study	Prediction of implantation outcome	NA	2,453 embryos	Naive Bayes classifier		Predict embryo implantation	Accuracy: 80.4%<br>Sensitivity: 63.7%<br>False alarm rate in embryo-based implantation prediction: 17.6%		
(Kaufmann *et al.*, 1997)	Retrospective study	Prediction of IVF outcome	Patients at their first IVF cycle	455 records (freezing, age, number of eggs recovered, number of embryos transferred)	Artificial neural network (ANN)		Clinical pregnancy rate	Accuracy: 59%		
(Vogiatzi *et al.*, 2019)	Retrospective study	Prediction of IVF outcome	257 infertile couples	426 IVF/ICSI cycles	Artificial neural network (ANN)		Predict live birth outcome	During cross-validation, the system exhibited the following:<br>Sensitivity: 69.2%±2.36%,<br>Specificity: 69.19%±2.8% (OR 5.21±1.27)<br>PPV 36.96±3.44<br>NPV 89.61±1.09<br>OA: 69.19%±2.69%		
(Shen *et al.*, 2022)	Retrospective study	Prediction of pregnancy rate	RIF patients	45,921 cycles (group A: 34,175 cycles with two embryos transferred; group	Machine learning algorithms (RF, GBDT, AdaBoost, and MLP)		Predict pregnancy rate	*GROUP A*<br>RF: AUC-ROC: 0.7856<br>AUC-PR: 0.7902		

				B: 11,746 cycles with only one embryo transferred)				GBDT AUC-ROC: 0.8066<br>AUC-PR: 0.8134<br>AdaBoost AUC-ROC: 0.8129<br>AUC-PR: 0.8206<br>MLP: AUC-ROC: 0.8139; AUC-PR: 0.8197)<br><br>*GROUP B*<br>RF: AUC-ROC: 0.8954<br>AUC-PR: 0.9003<br>GBDT AUC-ROC: 0.9025<br>AUC-PR: 0.9043<br>AdaBoost AUC-ROC: 0.9031<br>AUC-PR: 0.9114<br>MLP: AUC-ROC: 0.8969<br>AUC-PR: 0.9032		

PCOS

REFERENCES	STUDY DESIGN	TECHNIQUE	POPULATION	SAMPLE SIZE	INTERVENTION	COMPARISON	OUTCOME VARIABLES	RESULTS	MEAN DIFFERENCE	P
(Zad *et al.*, 2024) Gradient Boosted Trees (GBT) models had the highest performance	Retrospective study	Prediction of PCOS prior to clinical diagnosis	30,601 women aged 18-45 years		Machine learning algorithms (4 models both linear and nonlinear)		PCOS ICD-9 diagnosis	Parsimonious predictive model: *Model 1:* AUC (SD) of 82.3% (1.7) *Model 2:* AUC (SD) 77.6% (1.3) *Model 3: AUC* (SD) 77.4% (1.6) *Model 4:* AUC (SD) of 79.1% (1.1)		

Other

REFERENCES	STUDY DESIGN	TECHNIQUE	POPULATION	SAMPLE SIZE	INTERVENTION	COMPARISON	OUTCOME VARIABLES	RESULTS	MEAN DIFFERENCE	P
(Kuroda *et al.*, 2022)										
(Mercuri *et al.*, 2022)										
(Correia *et al.*, 2023)	Retrospective study	Oocytes selection	NA	311,237 patients	Machine learning model		Predict the optimal number of oocytes to expose to sperm, reducing the number of unused embryos created	Among cycles recommended to expose fewer than all oocytes, the median (IQR) numbers recommended for 1 live birth were 7 oocytes (7-8) for patients aged less than 32 years, 8 (7-8) for patients aged 32 to 34 years, and 9 (9-11) for patients aged 35 to 37 years.		
(Ozer *et al.*, 2023)	Retrospective study	Prediction of risk factors that cause first trimester pregnancy loss in good-quality frozen-thawed embryo transfer (FET) cycles	NA	3,805 good-quality FET cycles	Machine learning algorithm (random forest)		Predict risk factors for first trimester pregnancy loss	History of recurrent pregnancy loss increased first trimester pregnancy loss (OR: 7.729, 95% CI: 5.908–10.142, P=0.000).<br><br>BMI >30 increased first trimester pregnancy loss compared to <25 (OR: 1.418, 95% CI: 1.025-1.950, P=0.033)<br><br>Endometrial preparation with artificial cycle increased first trimester pregnancy loss compared to natural cycle (OR: 2.101, 95% CI: 1.630-2.723, P=0.000).		

								Female age between 35-37 increased first trimester pregnancy loss compared to <30 (OR: 1.617, 95% CI: 1.120-2.316, P=0.018),<br><br>Female age >37 increased first trimester pregnancy loss compared to <30 (OR: 2.286, 95% CI: 1.146-4,38, P=0.016).<br><br>The presence of PCOS increased first trimester pregnancy loss (OR: 1.693, 95% CI: 1.198-2.390, P=0.002).<br><br>The number of previous IVF cycles, which is >3, increased first trimester pregnancy loss compared to <3 (OR: 2.182, 95% CI: 1.708-2.790, P=0.000).		
(Cheredath *et al.*, 2023)	Retrospective study	Blastocyst derived metabolites	56 couples	Spent culture medium of day-5 blastocysts	Machine learning models		Predict the embryo implantation potential based on metabolite levels of spent culture medium of day-5 blastocysts	The spent culture medium of blastocysts that resulted in successful embryo implantation had significantly lower pyruvate (p<0.05) and threonine (p<0.05) levels compared to medium control but not compared to spent culture medium related to embryos that failed to implant		
(Liu *et al.*, 2023)	Retrospective study	Prediction of live birth	103 patients	1758 blastocysts	Convolutional neural network		Predict live birth	Model using blastocyst images:	95% CI: 0.65 – 0.70	

					(CNN)			AUC: 0.67<br><br>Model using blastocyst images and patient couple's clinical features where EM-status related features are excluded: AUC: 0.74<br><br>Model using blastocyst images and patient couple's clinical features where EM-status related features are included: AUC: 0.77	95% CI: 0.72 – 0.76<br>95% CI: 0.75–0.79	
(Louis *et al.*, 2023)	Retrospective study	Prediction of clinical pregnancy	NA	Transferred embryo images of 697 patients	Machine learning models (decision tree, random forest, and gradient boosting)		Predict clinical pregnancy based on patient's clinical characteristics	Decision tree AUC: 0.62<br>Random forest AUC: 0.58<br>Gradient boosting AUC: 0.63		
(Xu *et al.*, 2023)	Retrospective study	Prediction of PCOS	NA	11,720 ovarian stimulation cycles	Least Absolute Shrinkage and Selection Operator (LASSO) logistic regression		Predict PCOS based on body mass index (BMI), upper limit of menstrual cycle length (UML), serum AMH levels, and basal androstenedione (A4) levels.	PCOS-3<br>AUC: 0.841 (95% CI: 0.826, 0.856)<br><br>PCOS-4<br>AUC: 0.846 (95% CI: 0.812–0.875)		
(Fanton *et al.*, 2022b)	Retrospective study	Ovarian stimulation	NA	30,278 patients	Machine learning model		Predict the optimal day of trigger during ovarian stimulation	A set of interpretable machine learning models were developed using linear regression with follicle counts and estradiol levels. When using the model to make day-by-day predictions of trigger or continuing stimulation, possible early and late triggers were	The linear regression model for predicting MII outcomes on the day of trigger had a MAE of 2.87 oocytes and an R2 of 0.64 on the test data set, and the model for predicting the next-day MII outcomes had an MAE (mean absolute error) of 3.02 oocytes and an R2 of 0.62 on the	

								identified in 48.7% and 13.8% of cycles, respectively. After propensity score matching, patients with early triggers had on average 2.3 fewer MII oocytes, 1.8 fewer 2PNs, and 1.0 fewer usable blastocysts compared with matched patients with on-time triggers, and patients with late triggers had on average 2.7 fewer MII oocytes, 2.0 fewer 2PNs, and 0.7 fewer usable blastocysts compared with matched patients with on-time triggers.	test data set. The next-day E2 levels were predicted with a MAE of 274 pg/mL and R2 of 0.88. Implementation of the follicle imputation algorithm improved the MAE by 0.09 oocytes and R2 by 0.02	
(Wang *et al.*, 2022)	Retrospective study		NA	24,730 patients			Predict clinical pregnancy based on patient's clinical features	The ovarian stimulation protocol is the most important factor affecting pregnancy outcomes. Long and ultra-long protocols have shown positive effects on clinical pregnancy among all protocols. Furthermore, total frozen and transferred embryos are positive for a clinical pregnancy, but female age and duration of infertility have negative effects on clinical pregnancy.		
(Chapron *et al.*, 2022)	Case-control study	Prediction of endometriosis risk	NA	2,527 (1,195 patients with histologically	Multiple regression model to devise a score		Predict risk of endometriosis	Score 1 C-index: 0.81 (95% CI: 0.79-0.83).		

				proven endometriosis = study group; 1332 patients without endometriotic lesions during surgery = study group)			based on patient questionnaire	Results for the three score 1 levels were:<br>≥25:<br>Specificity: 91% (95% CI: 89-93)<br><11:<br>Sensitivity: 91% (95% CI: 89-93)<br>≥18:<br>Specificity: 75% (95% CI: 72-78)<br>Sensitivity: 73% (95% CI: 70-76).<br>Score 2 C-index: 0.75 (95% CI: 73-77).<br>The three levels of score 2 were:<br>≥24:<br>Specificity: 82% (95% CI: 80-85)<br><7:<br>Sensitivity: 92% (95% CI: 90-94)<br>≥17:<br>Specificity: 62% (95% CI: 58-65)<br>Sensitivity: 78% (95% CI: 75-81).<br>The two scores were internally and externally validated.		
(McLernon *et al.*, 2022)	Retrospective study	Prediction of live birth rates	NA	88,613 patients	Logistic regression model		Predict the cumulative probability of a live birth over three complete IVF cycles (pretreatment model) and over the second or third complete IVF cycles in couples whose first full cycle had been unsuccessful. (posttreatment model)	The C-statistic for the pretreatment model was 0.71, increasing to 0.73 when AMH level was included in the model. For the posttreatment model, the C-statistic was 0.71.		
(Mehrjerd *et al.*, 2022a)	Retrospective study	Impact of endometrial thickness on the	NA	729 couples	Random forest model (RFM)		Predict ongoing pregnancy rate	79.4% in patients under IVF/ICSI treatment		

		ongoing pregnancy rate						73.5% in patients under IUI treatment.<br>In addition, cut-off points for endometrial thickness have been calculated in both treatments based on Odd Ratio (OR) with a 95% CI. Cut off points are obtained for IUI and IVF/ICSI treatment in 7.7 mm (OR: 1.6, p=.05, 95% CI: 1.08,2.7) and 9.99 mm (OR: 2.35, p=.03, 95% CI: 1.05,5.9) EMT, respectively.		
(Mehrjerd *et al.*, 2022b)	Retrospective study	Prediction of the success rate of infertility treatments	NA	2,485 IVF/ICSI/IUI cycles			Machine learning algorithms (MLP, Multi-Level Perceptron; SVM, Support Vector Machine; LR, Logistic Regression; RF, Random Forest; KNN, k-Nearest Neighbor; GNB, Gaussian Naïve Bayes)	MLP:<br>*IVF/ICSI*<br>AUC: 0.63<br>F1 score: 0.63<br>*IUI*<br>AUC: 0.68<br>F1 score: 0.75<br>SVM:<br>*IVF/ICSI*<br>AUC: 0.51<br>F1 score: 0.5<br>*IUI*<br>AUC: 0.64<br>F1 score: 0.75<br>LR:<br>*IVF/ICSI*<br>AUC: 0.69<br>F1 score: 0.60<br>*IUI*<br>AUC: 0.68<br>F1 score: 0.77<br>RF:<br>*IVF/ICSI*<br>AUC: 0.73<br>F1 score: 0.73<br>*IUI*<br>AUC: 0.70<br>F1 score: 0.8<br>KNN:<br>*IVF/ICSI*<br>AUC: 0.50<br>F1 score: 0.71		

								IUI<br>AUC: 0.64<br>F1 score: 0.78<br>GNB:<br>*IVF/ICSI*<br>AUC: 0.67<br>F1 score: 0.70<br>*IUI*<br>AUC: 0.67<br>F1 score: 0.75		
(Yang *et al.*, 2022)	Retrospective study	Prediction of pregnancy and live birth rates	NA	Morphokinetic data of 367 embryos (training set: 70%; test set: 30%)	Random forest learning algorithm		Predict pregnancy rate based on embryo morphokinetic data	AUC: 0.69		
							Predict live birth rate	AUC: 0.64		
(Ata *et al.*, 2021)	Retrospective study	Diagnosis of recurrent implantation failure			Mathematical model		Calculate the required number of blastocysts transferred to a woman to achieve a predefined cumulative probability of implantation under the assumption that embryo aneuploidy is the sole reason for implantation failure.<br>Calculate the total number of blastocysts transferred to a woman that would be required to provide a predefined cumulative probability of implantation under the assumption that all implantation failures would be solely because of EA.			
(Liu *et al.*, 2021)	Retrospective study	Prediction of embryo transfer outcomes	NA	401 patients	Machine learning algorithms		Predict clinical pregnancy based on patient's clinical features	Logistic regression AUC: 0.603<br>Support Vector Machine AUC: 0.554		

								Conditional Interference Tree AUC: 0.540 Random Forest AUC: 0.613		
(Kozar *et al.*, 2021)	Retrospective study	Prediction of artificial intrauterine insemination procedure outcome	NA	413 couples	Machine learning algorithms		Predict clinical outcome of artificial intrauterine insemination based on patient's clinical data	*Random forest model*: AUC: 0.66 Sensitivity: 0.432 Specificity: 0.756 *Partial least squares*: AUC: 0.62 Sensitivity: 0.459 Specificity: 0.734		
(Srivastava *et al.*, 2021)		Follicle tracking	26 patients	96 volume pairs	Unsupervised Deep Learning		Predict the quality and quantity of the follicular pool based on 3D transvaginal ultrasound			
(Ferrick *et al.*, 2020)	Retrospective study	Blastocyst metabolism and biomarkers of embryo viability	50 women	209 preimpantation embryos (fresh and frozen cycles)				Morphokinetics, morphology (Gardner grade), KIDScore, artificial intelligence grade (EmbryoScore), glucose and amino acid metabolism, and clinical pregnancies	Glucose consumption was at least 40% higher in blastocysts deemed of high developmental potential using either the Gardner grade (P<0.01, n=209), KIDScore (P<0.05, n=207) or EmbryoScore (P<0.05, n=184), compared to less viable blastocysts and in blastocysts that resulted in a clinical pregnancy compared to those that failed to implant (P<0.05, n=37). Additionally, duration of cavitation was inversely related to glucose consumption (P<0.05, n=200). Total amino acid consumption was significantly higher	

									in blastocysts with an EmbryoScore higher than the cohort median score (P<0.01, n=185). Furthermore, the production of amino acids was significantly lower in blastocysts with a high Gardner grade (P<0.05, n=209), KIDScore (P<0.05, n=207) and EmbryoScore (P<0.01, n=184).	
(Goyal *et al.*, 2020)	Retrospective study	Prediction of live-birth occurrence after the complete IVF cycle	NA	141,160 patient records	Machine Learning models (Multi-layer perceptron, K Nearest Neighbours, decision tree); deep learning model (DL Classifier); ensemble models (voting-hard classifier, random forest, AdaBoost)		Predict live birth occurence based on the patient's clinical parameters	Multi-layer perceptron<br>AUC: 77.9%<br>F1 score: 72.98%<br>Recall: 72%<br>Precision: 74%<br><br>K Nearest Neighbours<br>AUC: 77.6%<br>F1 score: 71%<br>Recall: 71%<br>Precision: 71%<br><br>Decision tree<br>AUC: 83.3%<br>F1 score: 76%<br>Recall: 76%<br>Precision: 76%<br><br>DL Classifier<br>AUC: 78%<br>F1 score: 72.49%<br>Recall: 72%<br>Precision: 73%<br><br>voting-hard classifier<br>AUC: 73.1%<br>F1 score: 73.98%<br>Recall: 73%<br>Precision: 75%<br><br>Random forest<br>AUC: 84.60%<br>F1 score: 76.49%<br>Recall: 76%<br>Precision: 77%<br><br>AdaBoost<br>AUC: 77.4%<br>F1 score: 72.98%		

								Recall: 72%<br>Precision: 74%		
(Letterie and Mac Donald, 2020)	Retrospective study	Ovarian stimulation	NA	2,603 cycles (autologous: 1,853; donor cycles: 750)	Machine learning algorithm		Predict four clinical decisions during ovarian stimulation: [1] stop stimulation or continue stimulation. If the decision was to stop, then the next automated decision was to [2] trigger or cancel. If the decision was to return, then the next key decisions were [3] number of days to follow-up and [4] whether any dosage adjustment was needed.	Algorithm accuracies for these four decisions are as follows: continue or stop treatment: 0.92; trigger and schedule oocyte retrieval or cancel cycle: 0.96; dose of medication adjustment: 0.82; and number of days to follow-up: 0.87. These accuracies are for first iteration of the algorithm.		
(Liao *et al.*, 2020)	Retrospective study	Development of a dynamic diagnosis grading system for infertility	NA	95,868 medical records of 60,648 couples with infertility	Random forest algorithm		Define a dynamic grading system for patients with infertility based on: age, body mass index, follicle-stimulating hormone level, antral follicle count, anti-Mullerian hormone level, number of oocytes, and endometrial thickness.	The grading system divided the condition of the patient with infertility into 5 grades from A to E. The worst E grade represented a 0.90% pregnancy rate, and the pregnancy rate in the A grade was 53.82%. The cross-validation results showed that the stability of the system was 95.94% (95% CI: 95.14%-96.74%).		
(Raef *et al.*, 2020)	Retrospective study	Prediction of embryo implantation	500 patients	1,360 transferred embryos	Machine learning algorithms (NB, naive bayes; SVM, support vector machine; NN, neural network; RF, random forest; KNN, k-nearest neighbor; DT, decision tree)		Predict embryo transfer outcome based on patient-related and ART cycle features	*AUC:*<br>NB: 88.9<br>SVM: 86.8<br>NN: 93.47<br>RF: 93.74<br>KNN: 92.14<br>DT: 89.14<br>*Accuracy*:<br>NB: 83<br>SVM: 80.2<br>NN: 90.4		

								RF: 90.4<br>KNN: 83.8<br>DT: 88.6		
(Tarín *et al.*, 2020)	Retrospective study	Prognostic model for women's assisted fecundity	NA	708 patients (women who had a live birth event in the first autologous IVF/ICSI cycle: 458; women who failed to have a live birth event after completing three autologous cycles IVF/ICSI cycles: 250)			Predict women's assisted fecundity	The value of the c-statistic was 0.718 (asymptotic 95% CI: 0.672-0.763) in the development set and 0.649 (asymptotic 95% CI: 0.560-0.738) in the validation set. The model adequately fitted the data with no significant over or underestimation of predictor effects.		
(Xu *et al.*, 2019)	Prospective observational cohort study	Distinction between ongoing pregnancy and non-ongoing pregnancy in early gestational age	NA	1,650 fresh embryo transfer cycles (training set: 70%; test set: 30%)	Computational model (logistic regression)		Predict pregnancy outocome based on hGC levels	AUC: 0.903		
(Burai *et al.*, 2018)		computer-aided tools for the extraction of uterus wall from video hysteroscopy		28 hysteroscopic videos	Fully convolutional neural networks (FCNNs)		Increase segmentation accuracy	91.56% segmentation accuracy regarding the recognition of the uterus wall. Experimental evaluation indicated 0.9156 Dice and 0.8443 Jaccard scores		
(Esteves *et al.*, 2019)	Retrospective study	ART calculator (number of oocytes)	NA	1,464 consecutive infertile couples			Number of Metaphase II Oocytes			
(Huang *et al.*, 2021b)	Retrospective study	Maternal Immune Environment for Predicting Pregnancy Outcomes		Women with recurrent reproductive failure	Deep learning algorithm			Deep Learning did not significantly enhance the effect on prediction.		
(Li *et al.*, 2021)	Retrospective study	Prediction of pregnancy failure	Patients with poor ovarian response (POR) from 2016 to 2019	281 patients (training group: 179; validation group: 102)	Nomogram		Predict the probability of clinical pregnancy failure	AUC<br>Training set: 0.786<br>Validation set: 0.748	95% CI: 0.710–0.861<br>95% CI: 0.668–0.827	
(Jin *et al.*, 2021)	Retrospective study	Prediction of blastulation rate	Patients With Tubal Factor Infertility, Polycystic Ovary Syndrome, or Endometriosis	6,938 patients (tubal factor group: training set: 3,403; validation set: 1,375) (PCOS group: training set: 1,128; validation	Nomogram		Predict the probability of blastulation rate	Tubal factor group<br>Training set: 0.740<br>Validation set: 0.735<br><br>PCOS group<br>Training set: 0.776	95% CI: 0.724 – 0.757<br>95% CI: 0.708 – 0.762<br><br>95% CI: 0.749 – 0.803	

				set: 491) (endometriosis group: training set: 363; validation set: 178)				Validation set: 0.787<br>Endometriosis group<br>Training set: 0.739<br>Validation set: 0.750	95% CI: 0.748 – 0.872<br>95% CI: 0.686 – 0.792<br>95% CI: 0.675 – 0.824	
(Leijdekkers *et al.*, 2018)		McLernon models, predicting cumulative live birth rates (LBR)			Are the published pre-treatment and post-treatment McLernon models, predicting cumulative live birth rates (LBR) over multiple complete IVF cycles, valid in a different context? With minor recalibration of the pre-treatment model, both McLernon models accurately predict cumulative LBR in a different geographical context and a more recent time period.					
(Wald *et al.*, 2005)	Retrospective study	Predict intrauterine pregnancies	NA	85 patients (22–43 years old, mean age 32.45 years), who had undergone 113 IVF/ICSI cycles, primarily for male factor infertility	4-hidden node neural network		Predict intrauterine pregnancies	ROC areas 0.923 and 0.783 for the training and test sets respectively		

FSH starting dose

REFERENCES	STUDY DESIGN	TECHNIQUE	POPULATION	SAMPLE SIZE	INTERVENTION	COMPARISON	OUTCOME VARIABLES	RESULTS	MEAN DIFFERENCE	P
(Kobanawa, 2023)	Retrospective study	Starting dose	NA		FSH dose calculator					
(Fanton *et al.*, 2022a)	Retrospective study	Starting dose	NA	18,591 patients	FSH dose calculator			Define the optimal starting dose of FSH using the K-nearest neighbours algorithm	30% of cycles were dose-responsive and 64% were flat-responsive. After propensity score matching, patients in the dose-responsive group who received an optimal starting dose of FSH had on average 1.5 more MII oocytes, 1.2 more 2PN embryos and 0.6 more usable blastocysts using 10 IU less of starting FSH and 195 IU less of total FSH compared with patients given non-optimal doses. In the flat-responsive group, patients who received a low starting dose of FSH had on average 0.3 more MII oocytes, 0.3 more 2PN embryos and 0.2 more usable blastocysts using 149 IU less of starting FSH and 1375 IU less of total FSH compared with patients with a high starting dose. The best performing KNN model was obtained using K=100 neighbours and the Manhattan	

									distance metric, where the similarity between any two patients was calculated using the sum of the absolute differences for each normalized input parameter. In terms of predicting MII oocytes, the KNN had a mean absolute error of 3.79 mature oocytes and an R^2 of 0.45.	
(Howles *et al.*, 2006)	Retrospective study	Starting dose	NA	1,378 normo-ovulatory patients treated with recombinant human follicle stimulating hormone (r-hFSH, follitropin alfa)	FSH dose calculator			Define the optimal starting dose of FSH based on a patient's baseline characteristics.	The strongest four independent factors that predicted a satisfactory ovarian response (at least 11 oocytes retrieved) were age, BMI, basal FSH and number of follicles<11 mm on baseline scan before pituitary down-regulation. A concordance probability index (C-index), i.e. the degree of association between the observed and the predicted dose according to the pre-defined predictive factors, was used to evaluate the model's ability to predict response. This C-index of 59.5%, based upon the four pre-defined factors of FSH level at screening, BMI, age and number of follicles < 11mm, predicts that	

									approximately 60% of cases will be dosed correctly to achieve a satisfactory ovarian response.	

References

Abbasi A, Miahi E, Mirroshandel SA. Effect of deep transfer and multi-task learning on sperm abnormality detection. *Computers in Biology and Medicine* 2021;**128**:104121.

Adolfsson E, Porath S, Andershed AN. External validation of a time-lapse model; a retrospective study comparing embryo evaluation using a morphokinetic model to standard morphology with live birth as endpoint. *JBRA Assist Reprod* 2018;**22**:205–214.

Alegre L, Del Gallego R, Arrones S, Hernández P, Muñoz M, Meseguer M. Novel noninvasive embryo selection algorithm combining time-lapse morphokinetics and oxidative status of the spent embryo culture medium. *Fertil Steril* 2019;**111**:918-927.e3.

Ata B, Kalafat E, Somigliana E. A new definition of recurrent implantation failure on the basis of anticipated blastocyst aneuploidy rates across female age. *Fertil Steril* 2021;**116**:1320–1327.

Bamford T, Easter C, Montgomery S, Smith R, Dhillon-Smith RK, Barrie A, Campbell A, Coomarasamy A. A comparison of 12 machine learning models developed to predict ploidy, using a morphokinetic meta-dataset of 8147 embryos. *Human Reproduction* 2023;**38**:569–581.

Bardet L, Excoffier J-B, Salaun-Penquer N, Ortala M, Pasquier M, Mathieu d'Argent E, Massin N. Comparison of predictive models for cumulative live birth rate after treatment with ART. *Reproductive BioMedicine Online* 2022;**45**:246–255.

Barnes J, Brendel M, Gao VR, Rajendran S, Kim J, Li Q, Malmsten JE, Sierra JT, Zisimopoulos P, Sigaras A, *et al.* A non-invasive artificial intelligence approach for the prediction of human blastocyst ploidy: a retrospective model development and validation study. *The Lancet Digital Health* 2023;**5**:e28–e40.

Blais I, Koifman M, Feferkorn I, Dirnfeld M, Lahav-Baratz S. Improving embryo selection by the development of a laboratory-adapted time-lapse model. *F S Sci* 2021;**2**:176–197.

Bori L, Paya E, Alegre L, Viloria TA, Remohi JA, Naranjo V, Meseguer M. Novel and conventional embryo parameters as input data for artificial neural networks: an artificial intelligence model applied for prediction of the implantation potential. *Fertil Steril* 2020;**114**:1232–1241.

Burai P, Hajdu A, Manuel F-RE, Harangi B. Segmentation of the uterine wall by an ensemble of fully convolutional neural networks. *Annu Int Conf IEEE Eng Med Biol Soc* 2018;**2018**:49–52.

Canosa S, Licheri N, Bergandi L, Gennarelli G, Paschero C, Beccuti M, Cimadomo D, Coticchio G, Rienzi L, Benedetto C, *et al.* A novel machine-learning framework based on early embryo morphokinetics identifies a feature signature associated with blastocyst development. *J Ovarian Res* 2024;**17**:63.

Chapron C, Lafay-Pillet M-C, Santulli P, Bourdon M, Maignien C, Gaudet-Chardonnet A, Maitrot-Mantelet L, Borghese B, Marcellin L. A new validated screening method for endometriosis diagnosis based on patient questionnaires. *EClinicalMedicine* 2022;**44**:101263.

Cheredath A, Uppangala S, C S A, Jijo A, R VL, Kumar P, Joseph D, G A NG, Kalthur G, Adiga SK. Combining Machine Learning with Metabolomic and Embryologic Data Improves Embryo Implantation Prediction. *Reprod Sci* 2023;**30**:984–994.

Correia KFB, Missmer SA, Weinerman R, Ginsburg ES, Rossi BV. Development of a Model to Estimate the Optimal Number of Oocytes to Attempt to Fertilize During Assisted Reproductive Technology Treatment. *JAMA Netw Open* 2023;**6**:e2249395.

Coticchio G, Fiorentino G, Nicora G, Sciajno R, Cavalera F, Bellazzi R, Garagna S, Borini A, Zuccotti M. Cytoplasmic movements of the early human embryo: imaging and artificial intelligence to predict blastocyst development. *Reproductive BioMedicine Online* 2021;**42**:521–528.

Diakiw SM, Hall JMM, VerMilyea M, Lim AYX, Quangkananurug W, Chanchamroen S, Bankowski B, Stones R, Storr A, Miller A, *et al.* An artificial intelligence model correlated with morphological and genetic features of blastocyst quality improves ranking of viable embryos. *Reproductive BioMedicine Online* 2022;**45**:1105–1117.

Dirvanauskas D, Maskeliunas R, Raudonis V, Damasevicius R. Embryo development stage prediction algorithm for automated time lapse incubators. *Comput Methods Programs Biomed* 2019;**177**:161–174.

Esteves SC, Yarali H, Ubaldi FM, Carvalho JF, Bento FC, Vaiarelli A, Cimadomo D, Özbek İY, Polat M, Bozdag G, *et al.* Validation of ART Calculator for Predicting the Number of Metaphase II Oocytes Required for Obtaining at Least One Euploid Blastocyst for Transfer in Couples Undergoing in vitro Fertilization/Intracytoplasmic Sperm Injection. *Front Endocrinol (Lausanne)* 2019;**10**:917.

Fanton M, Nutting V, Rothman A, Maeder-York P, Hariton E, Barash O, Weckstein L, Sakkas D, Copperman AB, Loewke K. An interpretable machine learning model for individualized gonadotrophin starting dose selection during ovarian stimulation. *Reprod Biomed Online* 2022a;**45**:1152–1159.

Fanton M, Nutting V, Solano F, Maeder-York P, Hariton E, Barash O, Weckstein L, Sakkas D, Copperman AB, Loewke K. An interpretable machine learning model for predicting the optimal day of trigger during ovarian stimulation. *Fertil Steril* 2022b;**118**:101–108.

Ferrand T, Boulant J, He C, Chambost J, Jacques C, Pena C-A, Hickman C, Reignier A, Fréour T. Predicting the number of oocytes retrieved from controlled ovarian hyperstimulation with machine learning. *Human Reproduction* 2023;**38**:1918–1926.

Ferrick L, Lee YSL, Gardner DK. Metabolic activity of human blastocysts correlates with their morphokinetics, morphological grade, KIDScore and artificial intelligence ranking. *Hum Reprod* 2020;**35**:2004–2016.

Fu K, Li Y, Lv H, Wu W, Song J, Xu J. Development of a Model Predicting the Outcome of In Vitro Fertilization Cycles by a Robust Decision Tree Method. *Front Endocrinol* 2022;**13**:877518.

Fukunaga N, Sanami S, Kitasaka H, Tsuzuki Y, Watanabe H, Kida Y, Takeda S, Asada Y. Development of an automated two pronuclei detection system on time-lapse embryo images using deep learning techniques. *Reprod Medicine & Biology* 2020;**19**:286–294.

Geller J, Collazo I, Pai R, Hendon N, Lokeshwar SD, Arora H, Molina M, Ramasamy R. An Artificial Intelligence-Based Algorithm for Predicting Pregnancy Success Using Static Images Captured by Optical Light Microscopy during Intracytoplasmic Sperm Injection. *J Hum Reprod Sci* 2021;**14**:288–292.

Giscard d'Estaing S, Labrune E, Forcellini M, Edel C, Salle B, Lornage J, Benchaib M. A machine learning system with reinforcement capacity for predicting the fate of an ART embryo. *Syst Biol Reprod Med* 2021;**67**:64–78.

Gong X, Zhang Y, Zhu Y, Wang P, Wang Z, Liu C, Zhang M, La X. Development and validation of a live birth prediction model for expected poor ovarian response patients during IVF/ICSI. *Front Endocrinol* 2023;**14**:1027805.

Goyal A, Kuchana M, Ayyagari KPR. Machine learning predicts live-birth occurrence before in-vitro fertilization treatment. *Sci Rep* 2020;**10**:20925.

Howles CM, Saunders H, Alam V, Engrand P, FSH Treatment Guidelines Clinical Panel. Predictive factors and a corresponding treatment algorithm for controlled ovarian stimulation in patients treated with recombinant human follicle stimulating hormone (follitropin alfa) during assisted reproduction technology (ART) procedures. An analysis of 1378 patients. *Curr Med Res Opin* 2006;**22**:907–918.

Huang B, Tan W, Li Z, Jin L. An artificial intelligence model (euploid prediction algorithm) can predict embryo ploidy status based on time-lapse data. *Reprod Biol Endocrinol* 2021a;**19**:185.

Huang B, Zheng S, Ma B, Yang Y, Zhang S, Jin L. Using deep learning to predict the outcome of live birth from more than 10,000 embryo data. *BMC Pregnancy Childbirth* 2022;**22**:36.

Huang C, Xiang Z, Zhang Y, Tan DS, Yip CK, Liu Z, Li Y, Yu S, Diao L, Wong LY, *et al.* Using Deep Learning in a Monocentric Study to Characterize Maternal Immune Environment for Predicting Pregnancy Outcomes in the Recurrent Reproductive Failure Patients. *Front Immunol* 2021b;**12**:642167.

Javadi S, Mirroshandel SA. A novel deep learning method for automatic assessment of human sperm images. *Computers in Biology and Medicine* 2019;**109**:182–194.

Jiang A, Jiaqi W, Zhao H, Zhang Z, Sun Y. IDENTIFYING VIABILITY OF IMMOTILE SPERM AT ONE GLANCE: SPERM VIABILITY CLASSIFIER POWERED BY DEEP LEARNING. *Fertility and Sterility* 2022;**118**:e297–e298.

Jin H, Shen X, Song W, Liu Y, Qi L, Zhang F. The Development of Nomograms to Predict Blastulation Rate Following Cycles of In Vitro Fertilization in Patients With Tubal Factor Infertility, Polycystic Ovary Syndrome, or Endometriosis. *Front Endocrinol (Lausanne)* 2021;**12**:751373.

Jones CA, Christensen AL, Salihu H, Carpenter W, Petrozzino J, Abrams E, Sills ES, Keith LG. Prediction of individual probabilities of livebirth and multiple birth events following in vitro fertilization (IVF): a new outcomes counselling tool for IVF providers and patients using HFEA metrics. *J Exp Clin Assist Reprod* 2011;**8**:3.

Kanakasabapathy MK, Thirumalaraju P, Bormann CL, Kandula H, Dimitriadis I, Souter I, Yogesh V, Kota Sai Pavan S, Yarravarapu D, Gupta R, *et al.* Development and evaluation of inexpensive automated deep learning-based imaging systems for embryology. *Lab Chip* 2019;**19**:4139–4145.

Kaufmann SJ, Eastaugh JL, Snowden S, Smye SW, Sharma V. The application of neural networks in predicting the outcome of in- vitro fertilization. *Human Reproduction* 1997;**12**:1454–1457.

Kobanawa M. The gonadotropins starting dose calculator, which can be adjusted the target number of oocytes and stimulation duration days to achieve individualized controlled ovarian stimulation in Japanese patients. *Reprod Med Biol* 2023;**22**:e12499.

Kozar N, Kovač V, Reljič M. Can methods of artificial intelligence aid in optimizing patient selection in patients undergoing intrauterine inseminations? *J Assist Reprod Genet* 2021;**38**:1665–1673.

Kuroda S, Karna KK, Raneen Sawaid Kaiyal, Sajal Gupta, Rakesh Sharma, Agarwal A. DEVELOPMENT OF A NOVEL ROBUST ARTIFICIAL INTELLIGENCE DEVELOPED SPERM DNA FRAGMENTATION TEST – PRELIMINARY FINDINGS. *Fertility and Sterility* 2022;**118**:e307.

Lee C-I, Su Y-R, Chen C-H, Chang TA, Kuo EE-S, Zheng W-L, Hsieh W-T, Huang C-C, Lee M-S, Liu M. End-to-end deep learning for recognition of ploidy status using time-lapse videos. *J Assist Reprod Genet* 2021;**38**:1655–1663.

Leijdekkers JA, Eijkemans MJC, Tilborg TC van, Oudshoorn SC, McLernon DJ, Bhattacharya S, Mol BWJ, Broekmans FJM, Torrance HL, OPTIMIST group. Predicting the cumulative chance of live birth over multiple complete cycles of in vitro fertilization: an external validation study. *Hum Reprod* 2018;**33**:1684–1695.

Letterie G, Mac Donald A. Artificial intelligence in in vitro fertilization: a computer decision support system for day-to-day management of ovarian stimulation during in vitro fertilization. *Fertil Steril* 2020;**114**:1026–1031.

Letterie G, MacDonald A, Shi Z. An artificial intelligence platform to optimize workflow during ovarian stimulation and IVF: process improvement and outcome-based predictions. *Reproductive BioMedicine Online* 2022;**44**:254–260.

Li F, Lu R, Zeng C, Li X, Xue Q. Development and Validation of a Clinical Pregnancy Failure Prediction Model for Poor Ovarian Responders During IVF/ICSI. *Front Endocrinol (Lausanne)* 2021;**12**:717288.

Liao Q, Zhang Q, Feng X, Huang H, Xu H, Tian B, Liu J, Yu Q, Guo N, Liu Q, *et al.* Development of deep learning algorithms for predicting blastocyst formation and quality by time-lapse monitoring. *Commun Biol* 2021;**4**:415.

Liao S, Pan W, Dai W-Q, Jin L, Huang G, Wang R, Hu C, Pan W, Tu H. Development of a Dynamic Diagnosis Grading System for Infertility Using Machine Learning. *JAMA Netw Open* 2020;**3**:e2023654.

Liu H, Zhang Z, Gu Y, Dai C, Shan G, Song H, Li D, Chen W, Lin G, Sun Y. Development and evaluation of a live birth prediction model for evaluating human blastocysts from a retrospective study. *eLife***12**:e83662.

Liu L, Jiao Y, Li X, Ouyang Y, Shi D. Machine learning algorithms to predict early pregnancy loss after in vitro fertilization-embryo transfer with fetal heart rate as a strong predictor. *Computer Methods and Programs in Biomedicine* 2020;**196**:105624.

Liu R, Bai S, Jiang X, Luo L, Tong X, Zheng S, Wang Y, Xu B. Multifactor Prediction of Embryo Transfer Outcomes Based on a Machine Learning Algorithm. *Front Endocrinol (Lausanne)* 2021;**12**:745039.

Liu Y, Feenan K, Chapple V, Matson P. Assessing efficacy of day 3 embryo time-lapse algorithms retrospectively: impacts of dataset type and confounding factors. *Hum Fertil (Camb)* 2019;**22**:182–190.

Louis CM, Handayani N, Aprilliana T, Polim AA, Boediono A, Sini I. Genetic algorithm-assisted machine learning for clinical pregnancy prediction in in vitro fertilization. *AJOG Glob Rep* 2023;**3**:100133.

McCallum C, Riordon J, Wang Y, Kong T, You JB, Sanner S, Lagunov A, Hannam TG, Jarvi K, Sinton D. Deep learning-based selection of human sperm with high DNA integrity. *Commun Biol* 2019;**2**:250.

McLernon DJ, Raja E-A, Toner JP, Baker VL, Doody KJ, Seifer DB, Sparks AE, Wantman E, Lin PC, Bhattacharya S, *et al.* Predicting personalized cumulative live birth following in vitro fertilization. *Fertil Steril* 2022;**117**:326–338.

Mehrjerd A, Rezaei H, Eslami S, Khadem Ghaebi N. Determination of Cut Off for Endometrial Thickness in Couples with Unexplained Infertility: Trustable AI. *Stud Health Technol Inform* 2022a;**294**:264–268.

Mehrjerd A, Rezaei H, Eslami S, Ratna MB, Khadem Ghaebi N. Internal validation and comparison of predictive models to determine success rate of infertility treatments: a retrospective study of 2485 cycles. *Sci Rep* 2022b;**12**:7216.

Mendizabal-Ruiz G, Chavez-Badiola A, Aguilar Figueroa I, Martinez Nuño V, Flores-Saiffe Farias A, Valencia-Murilloa R, Drakeley A, Garcia-Sandoval JP, Cohen J. Computer software (SiD) assisted real-time single sperm selection associated with fertilization and blastocyst formation. *Reproductive BioMedicine Online* 2022;**45**:703–711.

Mercuri N, Fjeldstad J, Krivoi A, Meriano J, Nayot D. A NON-INVASIVE, 2-DIMENSIONAL (2D) IMAGE ANALYSIS ARTIFICIAL INTELLIGENCE (AI) TOOL SCORES MATURE OOCYTES AND CORRELATES WITH THE QUALITY OF SUBSEQUENT BLASTOCYST DEVELOPMENT. *Fertility and Sterility* 2022;**118**:e78–e79.

Milewski R, Kuczyńska A, Stankiewicz B, Kuczyński W. How much information about embryo implantation potential is included in morphokinetic data? A prediction model based on artificial neural networks and principal component analysis. *Adv Med Sci* 2017;**62**:202–206.

Morales DA, Bengoetxea E, Larrañaga P, García M, Franco Y, Fresnada M, Merino M. Bayesian classification for the selection of in vitro human embryos using morphological and clinical data. *Comput Methods Programs Biomed* 2008;**90**:104–116.

Ottl S, Amiriparian S, Gerczuk M, Schuller BW. motilitAI: A machine learning framework for automatic prediction of human sperm motility. *iScience* 2022;**25**:104644.

Ozer G, Akca A, Yuksel B, Duzguner I, Pehlivanli AC, Kahraman S. Prediction of risk factors for first trimester pregnancy loss in frozen-thawed good-quality embryo transfer cycles using machine learning algorithms. *J Assist Reprod Genet* 2023;**40**:279–288.

Peng T, Liao C, Ye X, Chen Z, Li X, Lan Y, Fu X, An G. Machine learning-based clustering to identify the combined effect of the DNA fragmentation index and conventional semen parameters on in vitro fertilization outcomes. *Reprod Biol Endocrinol* 2023;**21**:26.

Petersen BM, Boel M, Montag M, Gardner DK. Development of a generally applicable morphokinetic algorithm capable of predicting the implantation potential of embryos transferred on Day 3. *Hum Reprod* 2016;**31**:2231–2244.

Qiu J, Li P, Dong M, Xin X, Tan J. Personalized prediction of live birth prior to the first in vitro fertilization treatment: a machine learning method. *J Transl Med* 2019;**17**:317.

Qu P, Chen L, Zhao D, Shi W, Shi J. Nomogram for the cumulative live birth in women undergoing the first IVF cycle: Base on 26, 689 patients in China. *Front Endocrinol* 2022;**13**:900829.

Raef B, Maleki M, Ferdousi R. Computational prediction of implantation outcome after embryo transfer. *Health Informatics J* 2020;**26**:1810–1826.

Reignier A, Girard J-M, Lammers J, Chtourou S, Lefebvre T, Barriere P, Freour T. Performance of Day 5 KIDScore™ morphokinetic prediction models of implantation and live birth after single blastocyst transfer. *J Assist Reprod Genet* 2019;**36**:2279–2285.

Saiffe Farías AF, Sakkas D, Chavez-Badiola A, Ocali O, Mendizabal G, Valencia R, Valadez A, Hernandez MI, Drakeley AJ, Cohen J. SINGLE-SPERM MOTILITY ANALYSIS DURING ICSI USING AN ARTIFICIAL INTELLIGENCE SPERM IDENTIFICATION SOFTWARE (SID) AND CORRELATION WITH MORPHOLOGY. *Fertility and Sterility* 2022;**118**:e56–e57.

Shen L, Zhang Y, Chen W, Yin X. The Application of Artificial Intelligence in Predicting Embryo Transfer Outcome of Recurrent Implantation Failure. *Front Physiol* 2022;**13**:885661.

Srivastava D, Gupta S, Kudavelly S, K VS, Ga R. Unsupervised Deep Learning based Longitudinal Follicular Growth Tracking during IVF Cycle using 3D Transvaginal Ultrasound in Assisted Reproduction. *Annu Int Conf IEEE Eng Med Biol Soc* 2021;**2021**:3209–3212.

Strouthopoulos C, Anifandis G. An automated blastomere identification method for the evaluation of day 2 embryos during IVF/ICSI treatments. *Comput Methods Programs Biomed* 2018;**156**:53–59.

Tarín JJ, Pascual E, García-Pérez MA, Gómez R, Hidalgo-Mora JJ, Cano A. A predictive model for women's assisted fecundity before starting the first IVF/ICSI treatment cycle. *J Assist Reprod Genet* 2020;**37**:171–180.

Thirumalaraju P, Kanakasabapathy MK, Bormann CL, Gupta R, Pooniwala R, Kandula H, Souter I, Dimitriadis I, Shafiee H. Evaluation of deep convolutional neural networks in classifying human embryo images based on their morphological quality. *Heliyon* 2021;**7**:e06298.

Tran D, Cooke S, Illingworth PJ, Gardner DK. Deep learning as a predictive tool for fetal heart pregnancy following time-lapse incubation and blastocyst transfer. *Hum Reprod* 2019;**34**:1011–1018.

Uyar A, Bener A, Ciray HN. Predictive Modeling of Implantation Outcome in an In Vitro Fertilization Setting: An Application of Machine Learning Methods. *Med Decis Making* 2015;**35**:714–725.

Valiuškaitė V, Raudonis V, Maskeliūnas R, Damaševičius R, Krilavičius T. Deep Learning Based Evaluation of Spermatozoid Motility for Artificial Insemination. *Sensors (Basel)* 2020;**21**:72.

VerMilyea MD, Tan L, Anthony JT, Conaghan J, Ivani K, Gvakharia M, Boostanfar R, Baker VL, Suraj V, Chen AA, *et al.* Computer-automated time-lapse analysis results correlate with embryo implantation and clinical pregnancy: a blinded, multi-centre study. *Reprod Biomed Online* 2014;**29**:729–736.

Vogiatzi P, Pouliakis A, Siristatidis C. An artificial neural network for the prediction of assisted reproduction outcome. *J Assist Reprod Genet* 2019;**36**:1441–1448.

Wald M, Sparks AET, Sandlow J, Van-Voorhis B, Syrop CH, Niederberger CS. Computational models for prediction of IVF/ICSI outcomes with surgically retrieved spermatozoa. *Reprod Biomed Online* 2005;**11**:325–331.

Wang C-W, Kuo C-Y, Chen C-H, Hsieh Y-H, Su EC-Y. Predicting clinical pregnancy using clinical features and machine learning algorithms in in vitro fertilization. *PLoS One* 2022;**17**:e0267554.

Wen J-Y, Liu C-F, Chung M-T, Tsai Y-C. Artificial intelligence model to predict pregnancy and multiple pregnancy risk following in vitro fertilization-embryo transfer (IVF-ET). *Taiwanese Journal of Obstetrics and Gynecology* 2022;**61**:837–846.

Xi Q, Yang Q, Wang M, Huang B, Zhang B, Li Z, Liu S, Yang L, Zhu L, Jin L. Individualized embryo selection strategy developed by stacking machine learning model for better in vitro fertilization outcomes: an application study. *Reprod Biol Endocrinol* 2021;**19**:53.

Xu H, Feng G, Shi L, Han Y, Huang Q, Li R. PCOSt: A non-invasive and cost-effective screening tool for polycystic ovary syndrome. *Innovation (Camb)* 2023;**4**:100407.

Xu H, Wei Y, Yang R, Feng G, Tang W, Zhang H, He Y, Feng Y, Li R, Qiao J. Prospective observational cohort study: Computational models for early prediction of ongoing pregnancy in fresh IVF/ICSI-ET protocols. *Life Sci* 2019;**222**:221–227.

Yang L, Peavey M, Kaskar K, Chappell N, Zhu L, Devlin D, Valdes C, Schutt A, Woodard T, Zarutskie P, *et al*. Development of a dynamic machine learning algorithm to predict clinical pregnancy and live birth rate with embryo morphokinetics. *F S Rep* 2022;**3**:116–123.

Yi WJ, Park KS, Paick JS. Morphological classification of sperm heads using artificial neural networks. *Stud Health Technol Inform* 1998;**52 Pt 2**:1071–1074.

Zad Z, Jiang VS, Wolf AT, Wang T, Cheng JJ, Paschalidis IC, Mahalingaiah S. Predicting polycystic ovary syndrome with machine learning algorithms from electronic health records. *Front Endocrinol (Lausanne)* 2024;**15**:1298628.

Zhao M, Xu M, Li H, Alqawasmeh O, Chung JPW, Li TC, Lee T, Tang PM, Chan DYL. Application of convolutional neural network on early human embryo segmentation during in vitro fertilization. *J Cellular Molecular Medi* 2021;**25**:2633–2644.

Zieliński K, Pukszta S, Mickiewicz M, Kotlarz M, Wygocki P, Zieleń M, Drzewiecka D, Drzyzga D, Kloska A, Jakóbkiewicz-Banecka J. Personalized prediction of the secondary oocytes number after ovarian stimulation: A machine learning model based on clinical and genetic data. In Panchenko AR, editor. *PLoS Comput Biol* 2023;**19**:e1011020.
